# First evaluation of wearable radiation protection for human deep space exploration, as flown on Artemis I

**DOI:** 10.1126/sciadv.adz1892

**Published:** 2026-08-12

**Authors:** Jordan M. Houri, Thomas Berger, Ramona Gaza, Hesham Hussein, Shirit Schwarz, Gideon Waterman, Daniel Matthiä, Karel Marsalek, Bartos Przybyla, Joachim Aeckerlein, Maximilian Radenhaeuser, Moritz Kasemann, Kathleen Coderre, W. Paul Segars, Ehsan Samei, Anuj J. Kapadia, Kerry Lee, Diego Laramore, Stuart P. George, Edward Semones, Oren Milstein

**Affiliations:** ^1^ StemRad Inc., Tampa, FL, USA.; ^2^ German Aerospace Center (DLR), Institute of Aerospace Medicine, Radiation Biology Department, Cologne, Germany.; ^3^ Space Radiation Analysis Group, NASA Johnson Space Center (NASA-JSC), Houston, TX, USA.; ^4^ Leidos, Performance and Readiness Division, Houston, TX, USA.; ^5^ Lockheed Martin Space, Houston, TX, USA.; ^6^ StemRad Ltd., Tel Aviv, Israel.; ^7^ Israel Space Agency (ISA), Tel Aviv, Israel.; ^8^ Advanced Medical Physics, Inc., Houston, TX, USA.; ^9^ Lockheed Martin Advanced Technology Center, Palo Alto, CA, USA.; ^10^ Department of Radiology, Duke University, Durham, NC, USA.; ^11^ Oak Ridge National Laboratory, Oak Ridge, TN, USA.; ^12^ The Aerospace Corporation, Houston, TX, USA.

## Abstract

With preparations underway for extended-duration crewed deep space missions, the health risks of solar particle events (SPEs) to astronauts are becoming increasingly pertinent. To address this hazard, the AstroRad vest, a personal radiation shielding garment providing targeted organ protection, was tested during Artemis I. Two anthropomorphic female phantoms, equipped with internal and external passive and active dosimeters, were flown aboard the Orion spacecraft: one unshielded and the other wearing AstroRad. Inner Van Allen belt transit active dosimeter measurements were extrapolated to simulate SPE scenarios, predicting effective dose reductions of ∼60% for an August 1972–like SPE and nearly 40% for an October 1989–like SPE, varying slightly with anatomical model. Such reductions could spare astronauts the equivalent of up to 193 and 131 days of deep space radiation exposure, respectively. These findings demonstrate that wearable shielding such as AstroRad could serve as a vital element for safe and sustainable human deep space exploration.

## INTRODUCTION

More than 50 years after the final Apollo mission, NASA’s Artemis program has set the goal of returning humans to deep space, beginning with a human presence in lunar orbit with regular landings on the lunar surface and leading to the first human missions to Mars (*1*). Although this program represents a major step forward in human space exploration, the fact remains that the shielding provided by Earth’s atmosphere and magnetic field is not available beyond low Earth orbit (LEO) and astronauts will have to contend with exposure to the compositionally complex, spatially and temporally variable space radiation environment, introducing critical risks to crew health and the completion of mission objectives (*2*).

Among these risks, sporadic and unpredictable solar particle events (SPEs) are of particular concern for astronaut safety. SPEs result from solar eruptions that release an intense burst of radiation, consisting primarily of protons, with a monotonically decreasing energy spectrum. During an SPE, the flux of protons with energies of more than 10 MeV may increase by four to five orders of magnitude over periods of time ranging from several hours to a few days (*3*). In addition, these solar storms cannot be anticipated more than a few hours in advance (*4*). Despite this, because of the predominance of lower energy protons in typical SPE spectra, this radiation can be attenuated by realistic levels of material shielding, unlike the low-flux but more penetrating chronic background galactic cosmic radiation (GCR) (*5*). Although SPEs propagate outward from the Sun, the event-integrated flux becomes nearly isotropic beyond ∼1 astronomical unit (*6*), indicating that any radiation shielding should maximize solid angle of coverage.

While coating an entire spacecraft hull with a meaningful thickness of radiation shielding remains impractical due to mass constraints (*7*), concentrating an equivalent thickness of shielding around astronauts’ most radiation-sensitive organs and tissues in the form of a vest incurs a significantly lower mass penalty. Therefore, while the use of shielding materials such as high-density polyethylene (HDPE) has been extensively explored as environmental radiation protection in space (*8*–*11*), StemRad and Lockheed Martin worked to create a low-mass emergency countermeasure solution in the form of a personal radiation shielding garment, termed the “AstroRad vest,” intended to be worn by astronauts during SPEs in deep space, reducing both the risk of radiation-induced cancer and the already low probability of acute radiation syndrome (ARS). The AstroRad vest further minimizes the mass of radiation shielding through a targeted shielding architecture of a spatially variable thickness that is inversely related to the degree of self-shielding provided by the underlying tissue at each point, a technique first developed for personal wearable gamma radiation shielding on Earth (*12*). This additional optimization also serves to improve the ergonomics of the vest by decreasing shielding thickness near areas that do not require as much additional protection. The ergonomic aspect was previously evaluated by astronauts under microgravity conditions as part of the Comfort and Human Factors AstroRad Radiation Garment Evaluation (CHARGE) onboard the International Space Station (ISS; 2019 to 2022) (*13*). During an SPE, the AstroRad vest could be either deployed as a standalone countermeasure or used in combination with an onboard storm shelter, such as the one that can be configured inside of Orion, in which case wearing the vest would provide protection to a crewmember exiting the storm shelter to perform mission-critical tasks.

**Fig. 1.**
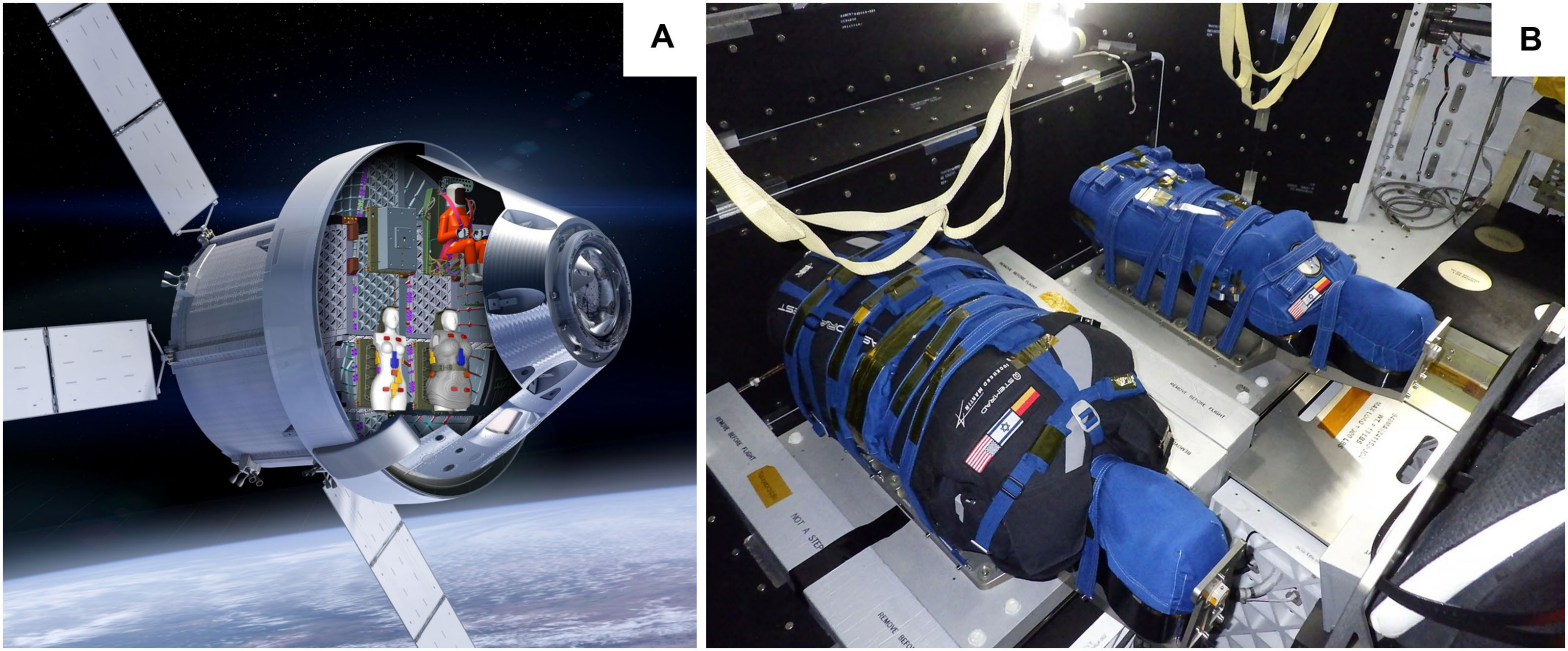
The Matroshka AstroRad Radiation Experiment. Illustration (**A**) and preflight photograph (**B**) of the MARE inside the Artemis I Orion spacecraft. Credit: NASA/Lockheed Martin/DLR.

This first test of the AstroRad vest’s radiation shielding efficacy in deep space took place during NASA’s uncrewed Artemis I lunar mission (November 16 to December 11 2022). In lieu of human crew, two identical tissue-equivalent radiotherapy-grade female torso dosimetry phantoms (“matroshkas”) were installed in seats 3 and 4 of the Orion spacecraft as part of the Matroshka AstroRad Radiation Experiment (MARE; Fig. 1). MARE was an international scientific collaboration co-led by the German Aerospace Center (DLR), the Israel Space Agency (ISA), and NASA and supported by StemRad and Lockheed Martin, building upon prior phantom studies in LEO (*14*–*24*) and heritage from European Space Agency (ESA) MATROSHKA (MTR) experiments on the ISS (*25*–*31*). Among these studies, MARE is the first experiment to measure the effect of wearable shielding on the deposition of radiation dose within a human phantom in space, as well as the first experiment in general to map the dose deposition within anthropomorphic phantoms beyond LEO. Female phantoms were chosen for this experiment due to females having larger risk of radiation-induced carcinogenesis (based on currently accepted risk models) (*32*–*34*). The phantom at seat 3, provided by ISA, was named Zohar and was fitted with the AstroRad radiation protection vest. The phantom at seat 4, provided by DLR, was named Helga and was not equipped with a vest, serving as the radiation exposure baseline (Helga was not an exact experimental control as the median aluminum shielding was 24.84 g/cm^2^ at seat 4 versus 24.40 g/cm^2^ at seat 3) (*35*, *36*).

As there were no SPEs during Artemis I, estimation of the AstroRad vest’s efficacy in its intended use case relied on model-based extrapolation of empirical data from the mission’s inner Van Allen belt transit. This was done by substituting the inner belt proton spectrum with models of the spectra of two major historical SPEs (August 1972 and October 1989). Estimating the dose distributions in this way (had such SPEs occurred during Artemis I) is made possible because the proton spectra within the Earth’s inner Van Allen belt exhibit a similar energy range and distribution as a typical SPE proton spectrum, albeit biased toward higher energies (i.e., more penetrating, due to the combined contribution of SPEs and cosmic ray albedo neutron decay to the trapped proton population; fig. S1) (*37*). Here, we present the outcomes of this analysis and discuss the implications of wearing a personal radiation shielding vest in deep space during a major SPE compared to remaining unshielded within the nominally configured Orion crew cabin (without the storm shelter).

## RESULTS

**Fig. 2.**
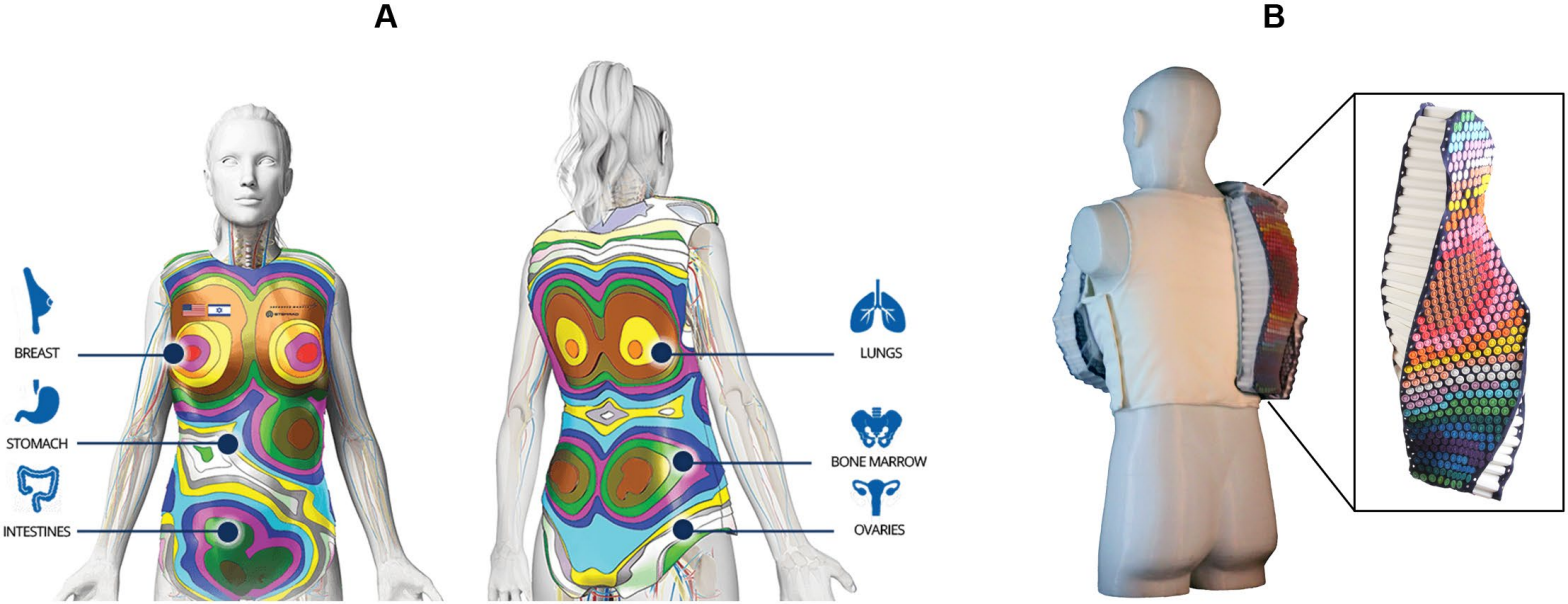
AstroRad vest design and internal structure. (**A**) AstroRad vest diagram with labeled target organs, adapted under license from 3dMediSphere/Shutterstock.com. (**B**) Section of an AstroRad shielding panel with tessellated hexagonal shielding components of varying thickness (denoted by color), demonstrating the flexibility of this structure.

The AstroRad vest used for the MARE experiment was custom-built for the Zohar phantom. In accordance with the 2007 Recommendations of the International Commission on Radiological Protection (ICRP), the vest optimizes protection for the red bone marrow (RBM), colon, lungs, stomach, breasts, and ovaries (Fig. 2A) (*38*). The shielding core of the MARE AstroRad vest is composed of HDPE, which has the largest percent composition by mass of hydrogen atoms of any nonvolatile and nontoxic material, maximizing the shielding efficacy against space radiation per unit mass as determined by the Bethe radiation stopping formula (*39*). Because HDPE is solid and inflexible, the shielding core consists of thousands of tessellated interconnected hexagonal rods, which behave in a scale-like manner to maximize flexibility, freedom of movement, and comfort (Fig. 2B). This design resulted in a vest with a mass of ∼26 kg.

**Fig. 3.**
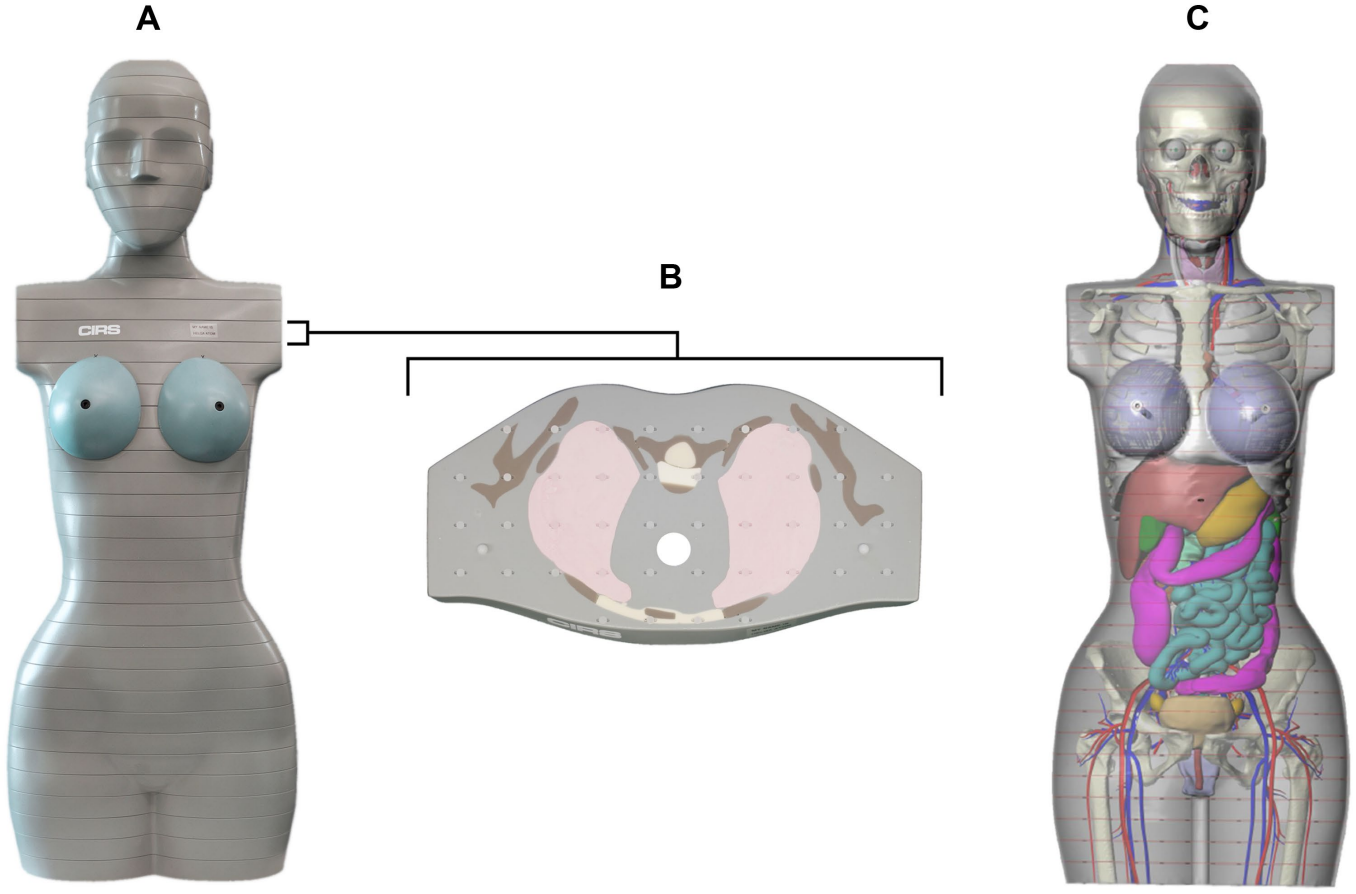
The MARE phantom. (**A**) Photo of the CIRS ATOM 702 Adult Female phantom, “Helga.” (**B**) Transverse slice from the Helga phantom exhibiting various tissues and organs (denoted by coloration) as well as the TLD grid. (**C**) Digital XCAT representation of the CIRS ATOM 702 Adult Female phantom.

The MARE anthropomorphic female torso phantoms were modified ATOM 702 Adult Female radiotherapy phantoms, manufactured by Computerized Imaging Reference Systems, Inc. (CIRS; Fig. 3A). These phantoms are constructed from 38 axial sections of height 25 mm and composed of CIRS proprietary tissue-equivalent resin representing soft tissue, average bone, cartilage, spinal cord, spinal disks, lung, brain, sinus, and breast (Fig. 3B). Organ locations and geometry (aside from organs and tissues that were already explicitly built in to the phantoms) were determined by performing computed tomography (CT) scans on each phantom and using those scans to generate a custom extended cardiac-torso (XCAT) digital phantom representation of the ATOM phantom, including 261 individual labeled anatomical structures corresponding to 22 different tissue types (Fig. 3C) (*40*, *41*).

**Fig. 4.**
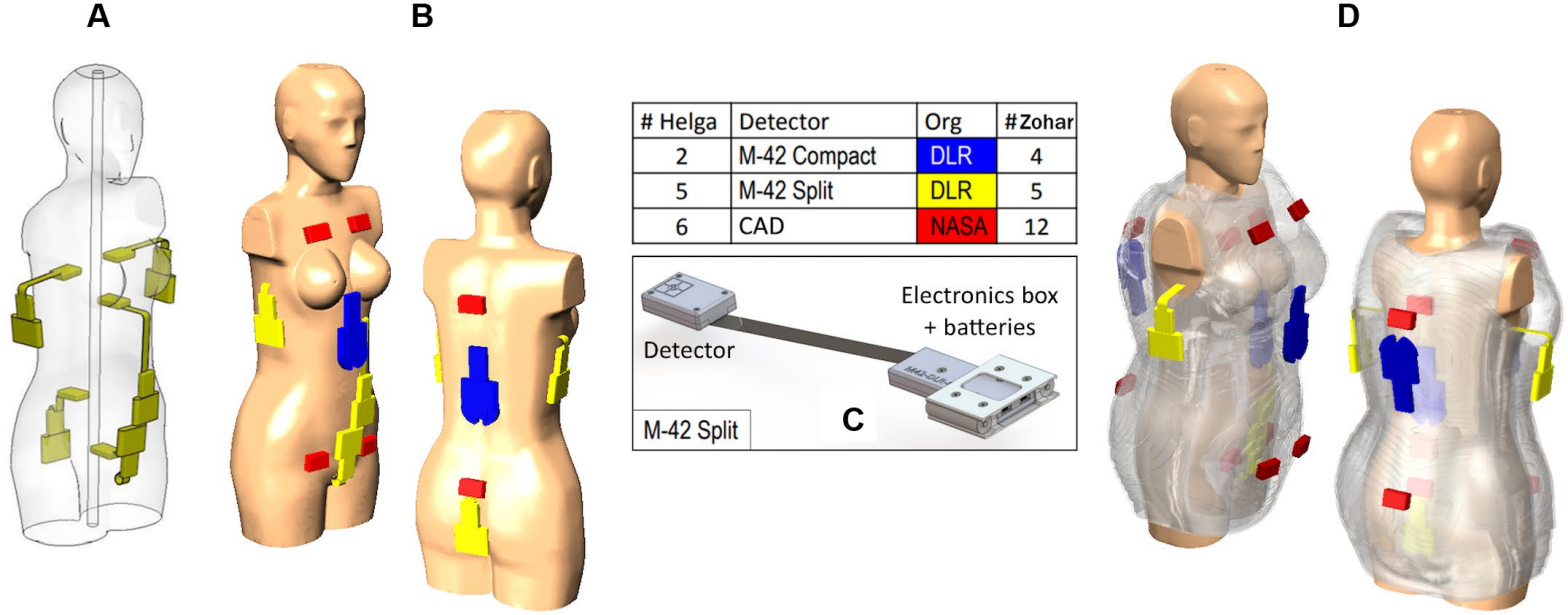
MARE active detector placement. (**A**) Internal-body view of the phantom showing the M-42 Split (internal) active detector placement for both Helga and Zohar (left/right lung, stomach, uterus, and spine). (**B**) External active detector placement for Helga showing the CADs (red), M-42 Compact detectors (blue), and the M-42 Split battery packs (yellow). (**C**) Diagram of the M-42 Split detector system clarifying the location of the detector, which is placed inside the phantom, and the battery pack, which hangs outside the phantom. (**D**) Active detector placement for Zohar (shown with a semitransparent AstroRad vest). The total number of active radiation detectors used across both phantoms was 34 (16 × DLR M-42 and 18 × NASA CAD).

Among the modifications to the standard ATOM 702 phantom, Helga and Zohar had cavities in the left and right lungs, stomach, uterus, and lower spinal region where internal-body DLR M-42 active dosimeters could be placed (Fig. 4, A and C) (*42*). For both Helga and Zohar, two additional external M-42 detectors were placed on the anterior and posterior surface, while for Zohar, a further two M-42s were placed on the anterior and posterior surface external to the AstroRad vest (Fig. 4, B and D). In addition, six NASA crew active dosimeters (CADs) were placed on the external surface of both Helga and Zohar at the upper front (anterior) left, upper front right, lower front left, lower front right, upper back (posterior) middle, and lower back middle (Fig. 4B) (*43*). A further six CADs were placed at the same locations on Zohar outside of the AstroRad vest (Fig. 4D).

### Validation of the MARE Monte Carlo simulation against Artemis I inner Van Allen belt transit data

**Fig. 5.**
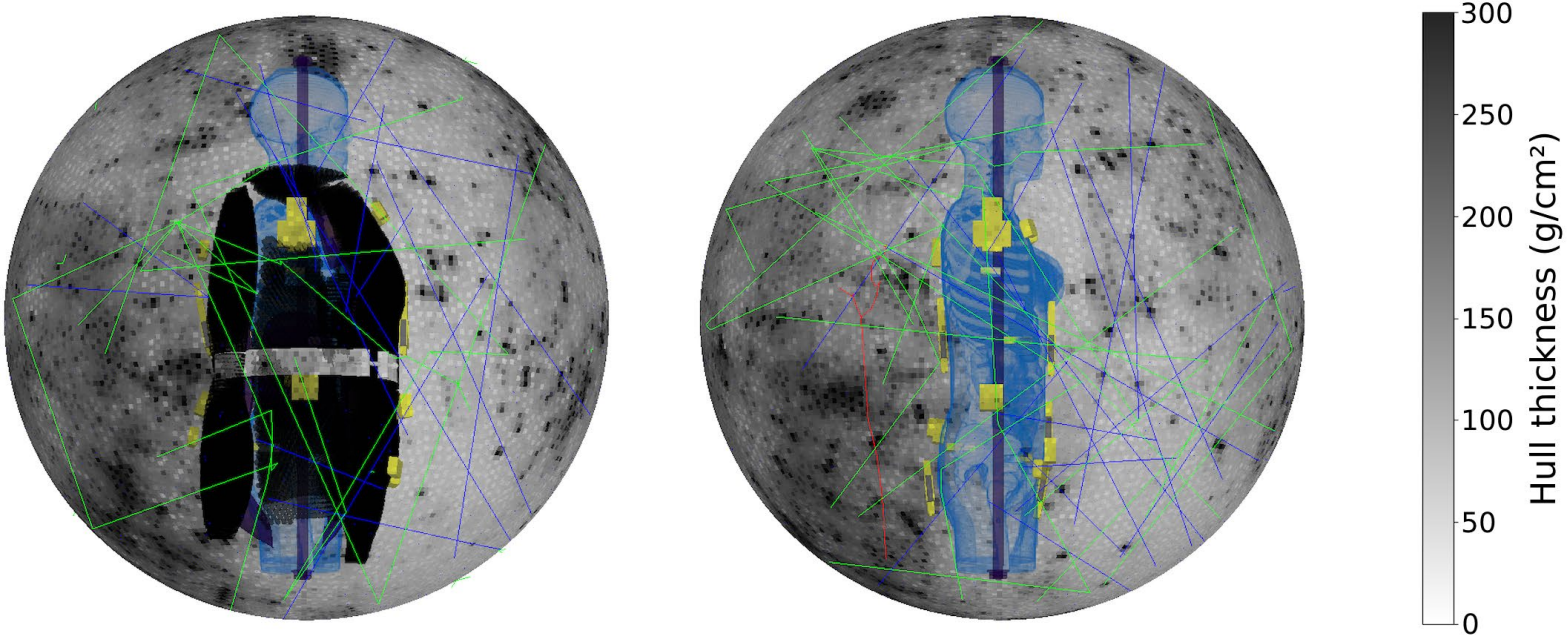
Validation/calibration phase simulation setup. GEANT4 visualization of the validation/calibration phase simulation setup for Zohar (left) and Helga (right) XCAT digital torso phantoms. Detectors are shown in yellow, PEEK support rod and Ultem dosimeter holder frames in purple, steel belt in gray, and AstroRad vest in black. The spacecraft hull is shown with darker shading denoting increased shielding thickness. Several simulated primary proton tracks are shown in blue, while secondary gammas and electrons are shown in green and red, respectively.

The M-42 and CAD active dosimeters each collected data during the inner Van Allen belt ascent phase of Artemis I, which served as the basis for extrapolation of results to hypothetical scenarios in which the Orion spacecraft were exposed to SPE radiation. This analysis was performed by developing and running mission-calibrated Monte Carlo radiation transport simulations using the GEANT4 platform for the Van Allen transit phase of the mission (*44*–*48*). The validation/calibration phase of the analysis centered around the development and execution of a Monte Carlo simulation intended to model the experimental conditions of Helga and Zohar’s journey through the inner Van Allen belt aboard Artemis I, with the objective of validating the simulation physics, geometry, and approximations via comparison of the simulated and experimental dose values recorded by the active detector systems on MARE. For these simulations, the Orion spacecraft hull for each phantom was modeled as a uniformly thick spherical shell with spatially varying mass density accounting for the directional distribution of areal thickness and material composition as viewed from either seat 3 or 4 in Orion (Fig. 5), generated by ray-tracing a manufacturing quality Orion CAD model in HZETRN (*49*). This method permits setting the uniform geometric thickness of the modeled hull to any arbitrary value (we set it to 1 cm for convenience of units), as the true areal thickness of the Orion hull was encoded entirely in the spatially varying mass density.

**Fig. 6.**
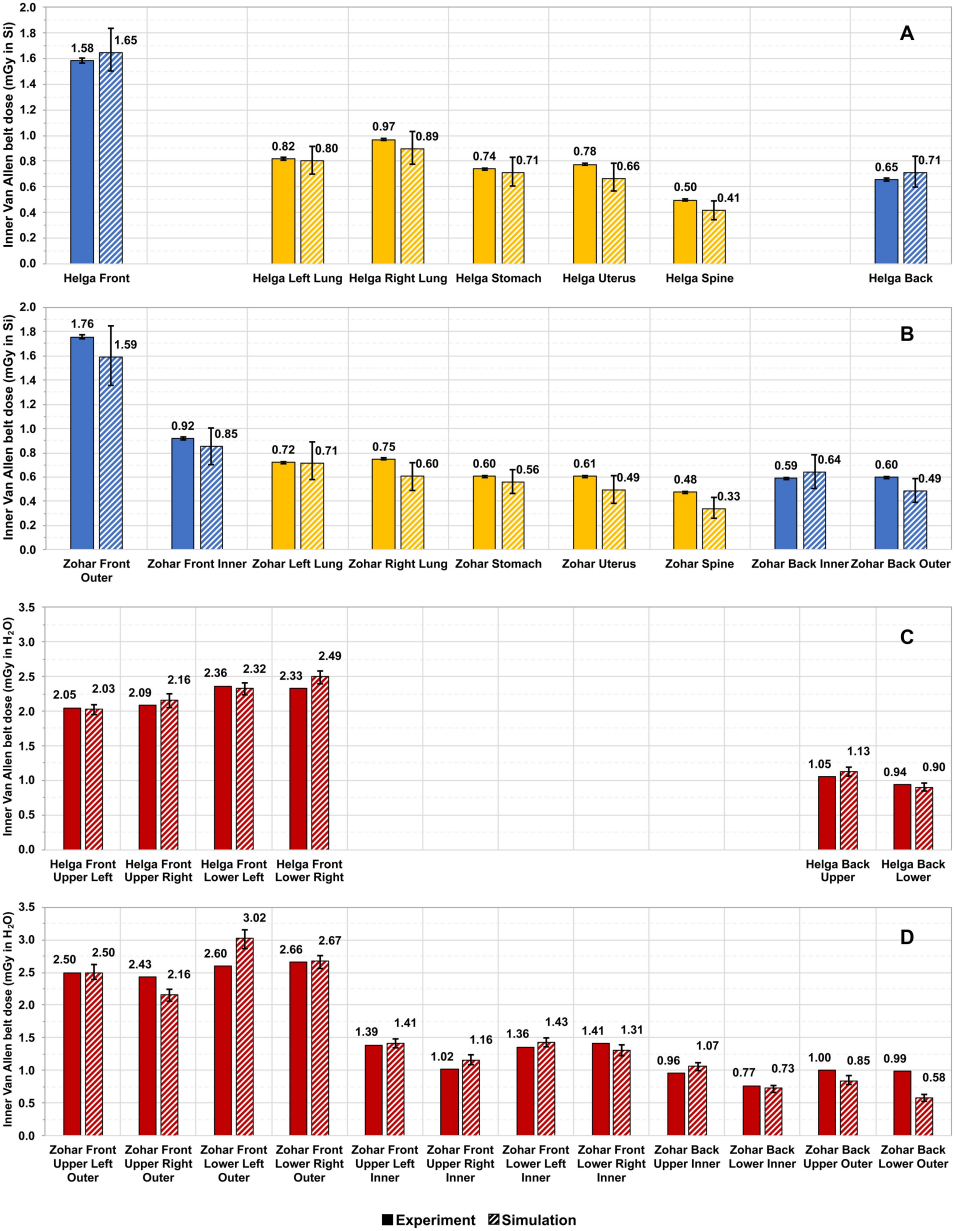
Simulation validation results. Comparison of simulated (hashed bars) versus actual (solid bars) M-42 Helga (**A**) and Zohar (**B**) and CAD Helga (**C**) and Zohar (**D**) dosimeter data for the Artemis I inner Van Allen belt crossing. Coloring corresponds to detector colors in Fig. 4, i.e., blue for external M-42s, yellow for internal M-42s, and red for CADs. Error bars represent 95% confidence intervals.

A comparison of cumulative dosimeter data (solid bars) as measured by the active dosimeters (DLR M-42s and NASA CADs) during the inner Van Allen belt transit of Artemis I with the corresponding results from Monte Carlo radiation transport simulations designed to accurately replicate the MARE experimental setup (hashed bars) is shown in Fig. 6. The simulated and measured dose values were very similar, with mean percent deviations of −4.6 ± 9.2% for the Helga M-42s (*N* = 7), −11.4 ± 11.1% for the Zohar M-42s (*N* = 9), 2.0 ± 4.7% for the Helga CADs (*N* = 6), and −3.0 ± 13.6% for the Zohar CADs (*N* = 12). In addition, the simulated and empirical datasets exhibit strong correlation, with the Pearson correlation coefficient *r* = 0.988 for the Helga M-42s, 0.984 for the Zohar M-42s, 0.993 for the Helga CADs, and 0.974 for the Zohar CADs.

**Fig. 7.**
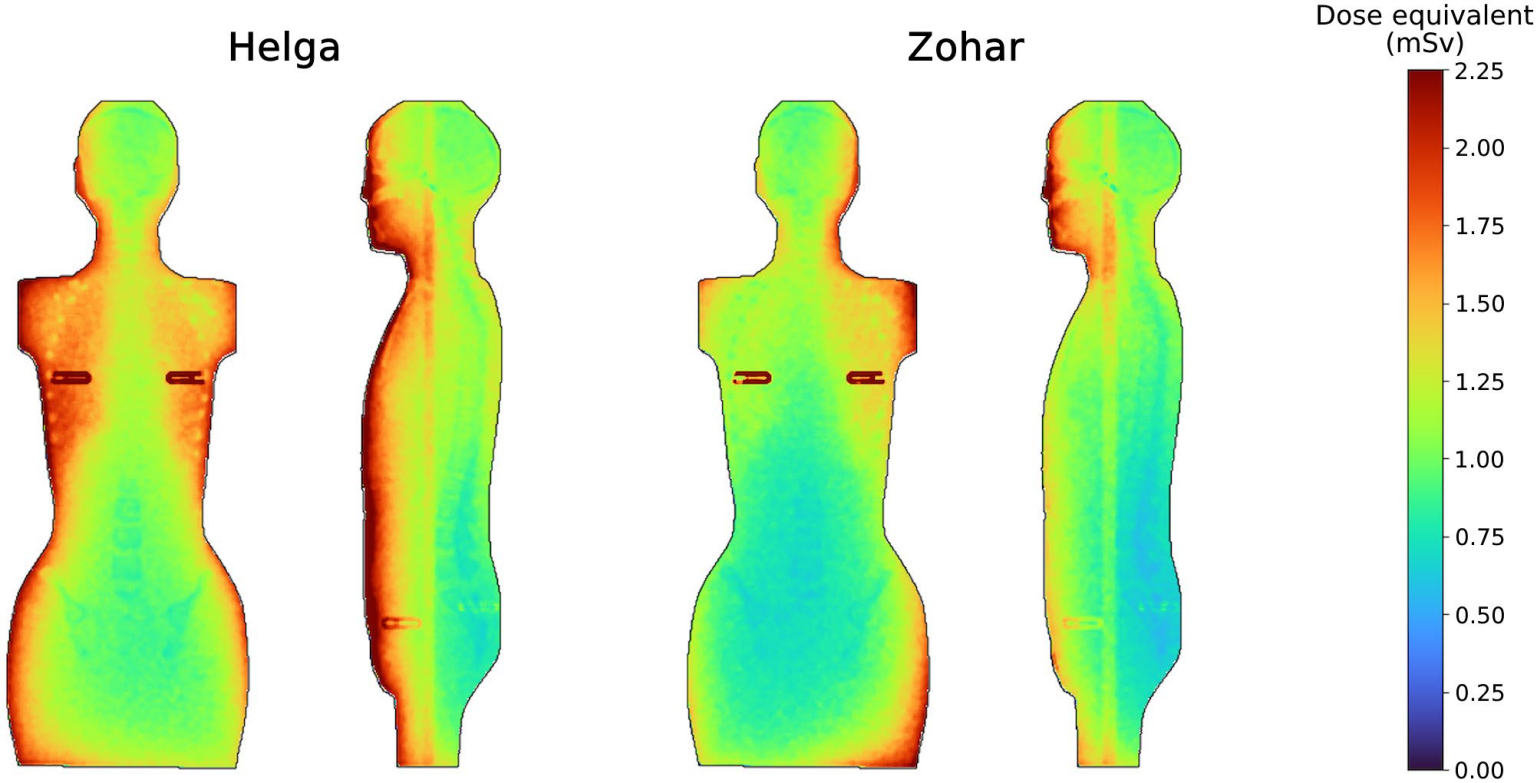
Simulated inner Van Allen belt dose maps for Helga and Zohar during Artemis I. Coronal and sagittal medial slices through the 3D NASA dose equivalent maps for Helga (left) and Zohar (right) obtained from the Artemis I inner Van Allen belt transit simulations. The dose hotspots in the lungs and anterior inferior body are due to the aluminum outer casings of the left and right lung and uterus M-42 detectors. These dose hotspots in aluminum do not significantly affect the dose readings by the silicon detectors within the aluminum casings. The vertical line with increased dose in the sagittal view is due to the PEEK support rod.

Coronal and sagittal cross-sections of the three-dimensional (3D) dose equivalent maps were calculated for both Helga and Zohar during the Artemis I inner Van Allen belt transit from the results of the validation/calibration simulations (Fig. 7). The shielding provided by the AstroRad vest, together with any local variations in spacecraft shielding (including slightly greater vehicle shielding at seat 3), in the relatively penetrating (compared to SPEs) proton environment of the inner Van Allen belt resulted in a total effective dose reduction of 25.8 ± 0.4% (from 1.61 ± 0.005 to 1.19 ± 0.006 mSv). Detailed dose values for specific tissues and organs are listed in Table 1.

**Table 1.** NASA dose equivalent for specific organs and tissues as calculated by the validation and SPE extrapolation simulations. Italicized values represent organs specifically targeted for protection by the AstroRad vest. For the SPE extrapolation simulations, results are provided for both the ATOM torso phantom and the whole-body phantom (in parentheses). This table is reproduced in table S2, with a color grading applied to the percent dose reduction columns.

	NASA dose equivalent (mSv)								
	Artemis I inner Van Allen belt transit			August 1972 SPE			October 1989 SPE		
	Helga	Zohar	Percent reduction	Helga or (whole-body unvested)	Zohar or (whole-body w/ AstroRad)	Percent reduction	Helga or (whole-body unvested)	Zohar or (whole-body w/ AstroRad)	Percent reduction
*Pelvic and lumbar red bone marrow*	*1.04*	*0.80*	*22.7*	*77.6 (72.4)*	*33.0 (30.9)*	*57.5 (57.3)*	*136.8 (130.8)*	*94.8 (90.9)*	*30.7 (30.5)*
*Breasts*	*2.52*	*1.55*	*38.4*	*510.2 (520.3)*	*127.8 (129.7)*	*75.0 (75.1)*	*398.2 (404.6)*	*179.0 (181.3)*	*55.0 (55.2)*
*Colon*	*1.60*	*1.09*	*32.0*	*153.4 (136.2)*	*53.3 (49.3)*	*65.3 (63.8)*	*187.8 (176.1)*	*114.6 (109.0)*	*39.0 (38.1)*
*Lungs*	*1.76*	*1.34*	*24.0*	*288.8 (245.0)*	*111.5 (95.5)*	*61.4 (61.0)*	*284.9 (254.5)*	*170.7 (153.5)*	*40.1 (39.7)*
*Gonads (ovaries and uterus)*	*1.27*	*0.97*	*23.5*	*53.7 (52.7)*	*26.6 (26.2)*	*50.5 (50.2)*	*119.0 (115.9)*	*88.6 (86.6)*	*25.6 (25.3)*
*Stomach*	*1.53*	*1.17*	*23.5*	*142.8 (122.2)*	*47.8 (43.2)*	*66.5 (64.7)*	*185.9 (167.4)*	*112.6 (106.1)*	*39.4 (36.6)*
Spine	0.96	0.81	15.6	115.7 (93.7)	62.6 (54.0)	45.9 (42.4)	167.1 (144.1)	120.6 (105.8)	27.8 (26.6)
Bladder	1.39	1.02	26.6	85.7 (82.7)	36.1 (33.5)	57.9 (59.5)	140.8 (136.0)	97.9 (95.6)	30.5 (29.7)
Liver	1.49	1.00	32.9	140.3 (120.9)	50.0 (43.2)	64.4 (64.3)	184.7 (168.7)	114.3 (105.2)	38.1 (37.6)
Esophagus	1.23	1.04	16.0	97.6 (84.5)	54.5 (48.3)	44.1 (42.9)	166.0 (149.8)	122.9 (111.9)	26.0 (25.3)
Thyroid	1.59	1.40	12.0	195.2 (158.6)	135.7 (114.3)	30.5 (28.0)	222.3 (189.5)	179.6 (163.5)	19.2 (13.7)
Skin	2.11	1.52	28.0	838.1 (708.4)	365.5 (508.0)	56.4 (28.3)	552.1 (494.4)	298.4 (382.4)	46.0 (22.7)
Bone surface	1.14	0.97	15.1	174.2 (172.9)	109.2 (151.8)	37.3 (12.2)	189.0 (195.8)	142.6 (176.7)	24.6 (9.8)
Salivary glands	1.72	1.59	7.8	294.5 (223.2)	239.2 (194.7)	18.8 (12.8)	272.2 (224.1)	236.7 (202.2)	13.0 (9.8)
Heart	1.38	1.07	22.2	103.1 (93.2)	48.1 (40.7)	53.4 (56.3)	166.6 (153.2)	116.1 (106.2)	30.3 (30.7)
Brain	1.17	1.14	2.4	171.3 (143.0)	165.9 (140.1)	3.1 (2.0)	204.3 (184.3)	198.0 (180.6)	3.1 (2.0)
Eyes	1.80	1.73	3.8	283.8 (235.7)	273.1 (229.3)	3.8 (2.7)	267.4 (234.0)	259.2 (228.2)	3.0 (2.5)
Intestines	1.48	1.07	27.9	124.8 (112.8)	44.4 (42.0)	64.4 (62.7)	170.3 (160.3)	106.6 (102.2)	37.4 (36.2)
Kidney	1.14	0.85	25.7	105.3 (97.4)	42.7 (40.5)	59.5 (58.5)	161.3 (154.1)	105.8 (100.5)	34.4 (34.8)
Blood and RBM (leukemia)	1.25	1.01	19.0	123.7 (105.9)	70.0 (64.6)	43.4 (39.0)	169.3 (154.6)	124.3 (116.8)	26.6 (24.5)
Oral cavity	1.68	1.59	5.2	192.6 (101.8)	175.0 (97.2)	9.1 (4.5)	220.8 (156.8)	203.3 (150.2)	7.9 (4.2)
Pancreas	1.24	0.93	25.5	65.6 (59.5)	30.1 (27.3)	54.1 (54.2)	136.8 (130.8)	95.4 (91.5)	30.3 (30.0)
Spleen	1.31	1.05	19.6	152.1 (135.3)	51.6 (49.5)	66.1 (63.4)	193.3 (179.4)	116.0 (110.7)	40.0 (38.3)
**Total NASA effective dose**	**1.61**	**1.19**	**25.8**	**222.3 (204.2)**	**87.5 (85.3)**	**60.7 (58.2)**	**233.5 (219.0)**	**143.6 (138.2)**	**38.5 (36.9)**

### Shielding effectiveness of the AstroRad vest inside the Orion spacecraft as exposed to historical SPEs

**Fig. 8.**
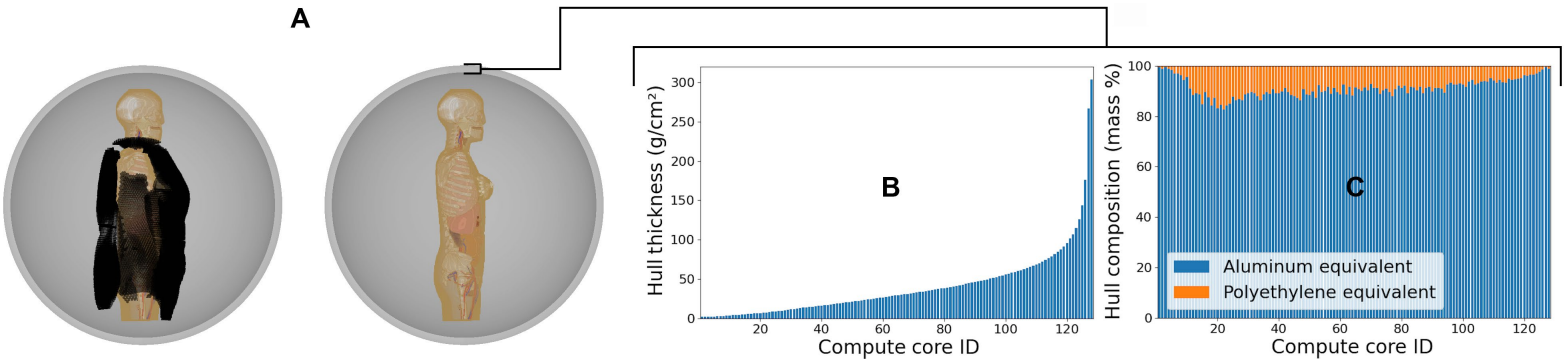
SPE extrapolation simulation setup with the ATOM torso phantom. (**A**) SPE extrapolation simulation setup visualized in GEANT4 with spherical hull of uniform thickness for Zohar (left, with AstroRad shown in black) and Helga (right) XCAT digital phantoms. (**B**) Distribution of hull thicknesses (in grams per square centimeter) versus compute core ID. (**C**) Hull material composition versus compute core ID.

Following validation of the GEANT4 model (which ultimately required no calibration, as explained in Discussion), the simulation setup was minimally modified to (i) eliminate any difference in hull shielding between the vested and unvested case, (ii) account for random rotation of phantoms within the spacecraft (as real astronauts would not be strapped to their seats in a forward-facing orientation during the full duration of an SPE), and (iii) simulate the occurrence of a major SPE similar to that of either August 1972 or October 1989. To simulate random rotation of the phantoms, the spacecraft hull was replaced with a spherical shell of directionally uniform thickness (in grams per square centimeter) and material composition (Fig. 8A), with these parameters varied across simulations running on different compute cluster cores, such that

$$f_{\left(g/{\text{cm}}^{2},\text{material composition}\right)}\left(\theta ,\varphi \right)\mapsto q_{\left(g/{\text{cm}}^{2},\text{material composition}\right)}\left(\text{CPU}\;\text{ID}\right)$$ (1)

where the directional distribution combines data points from seats 3 and 4 in Orion such that the vested and unvested phantoms experienced the same hull shielding distribution. The transformation in Eq. 1 maps the spatial distribution of hull shielding thickness and material composition, which depended on directional coordinates in the validation simulation, to a differential quantile function that depends on CPU core ID and should be interpreted to mean that, for instance, because 15.6% of the field of view from seats 3 and 4 of Orion is shielded by between 10 and 20 g/cm^2^, then 15.6% of computer cluster cores ran an iteration of the simulation with a uniform hull thickness within that range (Fig. 8, B and C). This method produces much more accurate results than would running a single simulation with a uniform, average-thickness hull, which would underestimate dose and the impact of additional shielding because dose is not linearly proportional to hull thickness and the least shielded parts of the spacecraft contribute disproportionately to dose (fig. S2). In addition to the implementation of rotational symmetry, these simulations were modeled with more accurate human tissue materials, rather than the tissue-equivalent resin materials used in the physical phantoms, and exclude MARE auxiliary hardware such as dosimeters and support structures.

**Fig. 9.**
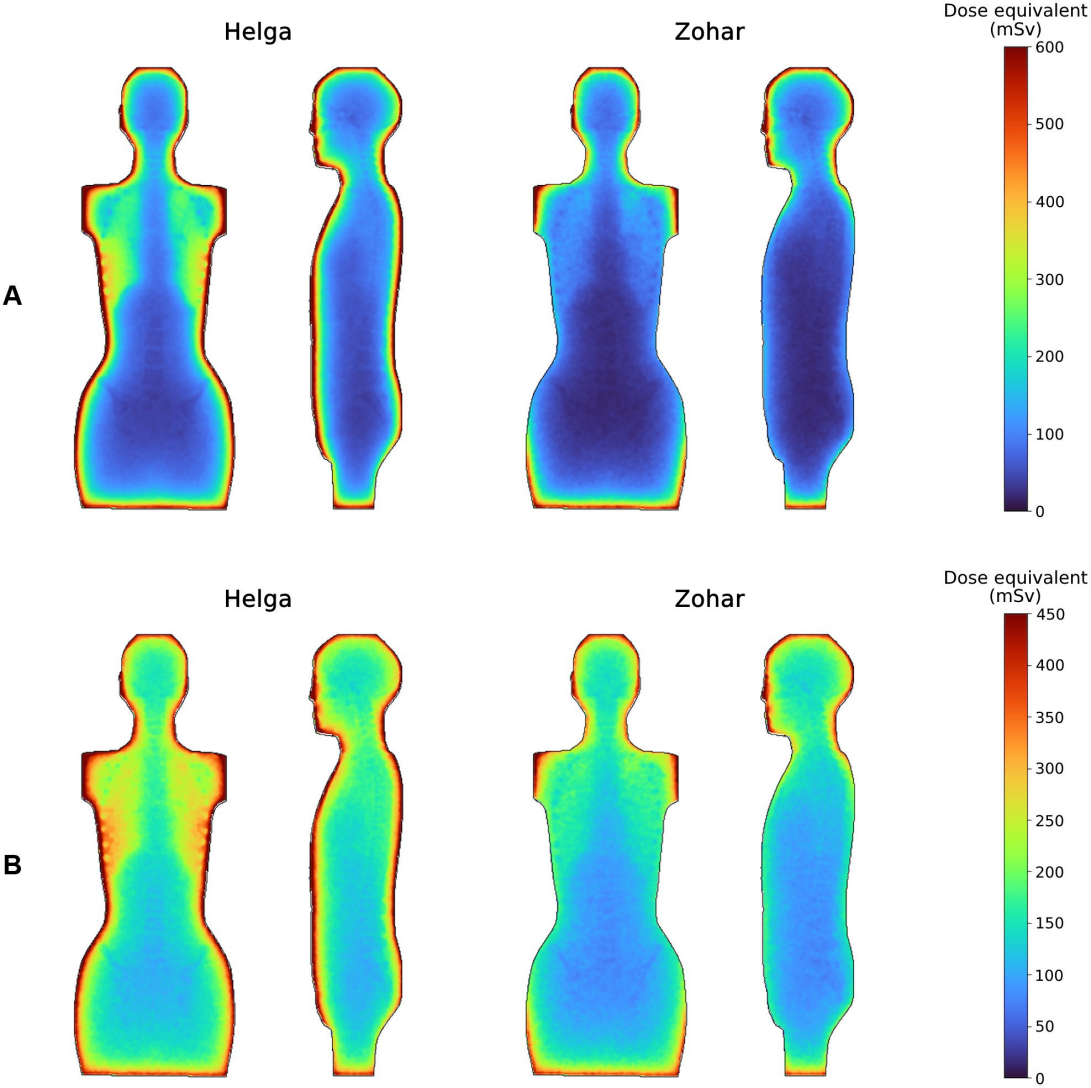
Dose maps with and without AstroRad resulting from the SPE extrapolation simulations using the ATOM torso phantom. Coronal and sagittal medial slices through the 3D NASA dose equivalent maps for Helga (left) and Zohar (right) as exposed to the August 1972 SPE (**A**) and the October 1989 SPE (**B**). Note the different color scale between the 1972 SPE and 1989 SPE dose maps.

The validated Monte Carlo dose predictions for Helga and Zohar, as exposed to historical proton spectra from the August 1972 SPE and the October 1989 SPE, have been graphically consolidated as coronal and sagittal cross-sections of the 3D dose equivalent maps (Fig. 9). For the August 1972 SPE spectrum, it was determined that the AstroRad vest would be expected to reduce the total effective dose from 222.3 to 87.5 mSv, a reduction of 60.7%. For the October 1989 SPE spectrum, it was determined that the AstroRad vest would be expected to reduce the total effective dose from 233.5 to 143.6 mSv, a reduction of 38.5%. Detailed dose values for specific tissues and organs for this set of simulations are also listed in Table 1. Table S2 reproduces Table 1 with a color grading applied to the percent dose reduction column.

A second set of SPE extrapolation simulations was performed in which the digital ATOM torso phantom representing Helga and Zohar was replaced with a whole-body median adult female XCAT digital phantom so as to evaluate the vest with a more realistic body geometry (fig. S3) (*40*, *41*). In this case, for the August 1972 SPE spectrum, it was determined that the AstroRad vest would be expected to reduce the total effective dose from 204.2 to 85.3 mSv, a reduction of 58.2%, whereas for the October 1989 SPE spectrum, it was determined that the vest would be expected to reduce the total effective dose from 219.0 to 138.2 mSv, a reduction of 36.9% (fig. S4, Table 1, and table S2 in parentheses).

Last, as a secondary measure of this study with the aim of evaluating the effectiveness of the targeted protection principle behind the design of the AstroRad vest, two smaller-scale simulations were run with a full body HDPE suit of uniform thickness and the same mass as the MARE AstroRad vest (16.4-mm thick, covering the entire body except the hands, feet, and face), also using the whole-body XCAT phantom (fig. S5). Compared to the whole-body simulations with the AstroRad vest, protection was reduced for all organs except the thyroid, skin, cortical bone, salivary glands, brain, eyes, and oral cavity (fig. S6 and table S3). Overall, the AstroRad vest using targeted shielding improved effective dose reduction by ∼30% compared with a uniform suit covering the entire body.

## DISCUSSION

This study evaluated the efficacy of radiation shielding by a personal protective vest, the AstroRad, within the Orion spacecraft crew cabin under conditions resembling the August 1972 and October 1989 SPEs. This was done by indirectly extrapolating empirical data from the MARE experiment onboard Artemis I (specifically, the active dosimeter data collected during the inner Van Allen belt transit) using Monte Carlo simulations.

The first phase of the analysis aimed to validate and/or calibrate the simulation physics and architecture via comparison of the simulated and measured active dosimeter results from the inner Van Allen belt transit stage of the mission. Because the AstroRad vest, MARE auxiliary hardware, and phantoms were all modeled with a high degree of geometric and compositional certainty, any potential differences between simulated and measured results were anticipated to stem from assumptions regarding the input radiation spectrum and spacecraft shielding. The shielding model, based on a raytracing analysis of a manufacturing-quality Orion CAD file, simplified the spacecraft hull to a blend of two materials (aluminum and polyethylene), which may have implications for the production of secondary radiation and does not account for shielding provided by many nonstructural objects inside the cabin. Furthermore, any potential scattering between adjacent angular bins in the segmented spherical hull model with spatially varying density (Fig. 5) could result in particle trajectories that might never occur in the real Orion geometry. The input radiation environment was calculated using the predictive AE9/AP9 trapped radiation computational models, which are climatological and may deviate from the true spectrum encountered by Orion during Artemis I (*50*). In addition, the AE9/AP9 models do not account for the directionality of the trapped radiation, which has been shown by a companion study using MARE data to have a nonnegligible impact on dose (*36*). Because it is impossible to know the degree to which each of these sources of uncertainty could affect model predictions, any deviations between the simulated and measured doses would have had to be accounted for via manual calibration via dose scaling. However, the “vanilla” model demonstrated strong agreement with experimental data: Correlation coefficients were near unity across all detector groupings (*R* = 0.993 for Helga CADs, *R* = 0.974 for Zohar CADs, *R* = 0.988 for Helga M-42s, and *R* = 0.984 for Zohar M-42s), with a mean percentage deviation in simulated dose across all detectors of −4.7% and no individual value exceeded an absolute percent deviation of 29.5%. As a result, the simulation was considered to be reliable, and no extra calibration procedures were performed.

The validation phase simulations also enabled comparison of the simulated 3D dose equivalent maps for Helga and Zohar during the Artemis I inner Van Allen belt passage, which is useful because such an analysis could not be performed directly using data from the passive thermoluminescence dosimeters (TLDs) embedded throughout each phantom, as these detectors only measured cumulative mission dose. From the estimated dose maps, it is evident that Helga had received a higher dose on the right side and Zohar had received a higher dose on the left side, which was expected, as those were the sides of the phantoms facing the exterior of the spacecraft. Increased anterior dose is clearly visible for both phantoms due to the large amount of aft shielding provided by the service module of the spacecraft. The AstroRad vest is not intended to be worn during the short passage through the Van Allen belts that would occur during most deep space missions, due mainly to the insignificant amount of dose that would be accumulated over such brief time scales, which has been shown here to be on the order of single-digit millisievert for an Artemis-class mission.

The outcomes of the SPE extrapolation simulations demonstrate substantial effectiveness of the AstroRad vest in terms of shielding astronauts against SPE radiation inside the Orion crew cabin. Despite the anatomical differences between the two phantom configurations (torso and whole-body), the percent effective dose reduction achieved by the vest was similar in each case, at ∼60% when exposed to a 1972-like SPE and in the range of 35 to 40% when exposed to a 1989-like SPE. The reduced effectiveness of the vest in the case of the October 1989 SPE spectrum is attributed to its higher energy (i.e., greater proton penetration power) than the August 1972 SPE. The October 1989 SPE may be considered as a worst-case scenario in terms of spectrum hardness, while the August 1972 SPE may be considered representative of a typical large SPE spectrum. Despite the similar dose reductions for each evaluated phantom configuration, raw effective doses were slightly higher for the ATOM torso phantoms than for the whole-body phantoms, which is primarily a consequence of the additional self-shielding provided by the limbs in the whole-body case.

With respect to the specific organ and tissue dose equivalent predictions, the reductions are once again found to be similar for the ATOM torso and whole-body phantoms. Notably, although there is a wide variation in dose to the AstroRad target organs in the unvested scenario, the doses to the AstroRad target organs are much more uniform under the protection of the vest, as expected due to the targeted shielding design. Furthermore, it is shown that, although the AstroRad vest does not provide direct coverage to organs such as the brain, eyes, oral cavity, and salivary glands, the presence of the vest slightly reduces dose to these organs as well, likely due to additional scattering and partial shielding by the vest mass from below.

The current NASA Space Flight Human-System Standard (NASA-STD-3001, Volume 1, Revision C) establishes a total career limit of 600 mSv effective dose for each astronaut, irrespective of age or gender, which maintains the estimated risk of exposure-induced death (REID) below 3% for all demographics (*51*). Therefore, it is expected, based on the results for the whole-body phantoms, that unshielded astronauts within the Orion crew cabin would receive ∼36.5% of their career dose limit if exposed to a single October 1989–like SPE and ∼34.0% of this limit if exposed to a single August 1972–like SPE, while wearing the AstroRad vest would reduce these percentages to ∼23.0 and ∼14.2%, respectively. By accounting for expected background GCR exposures in deep space along with the 600-mSv limit, it is possible to calculate how much of the useful spaceflight career of an astronaut may be preserved in such scenarios. The internal M-42 detectors (lungs, stomach, uterus, and spine) in Helga recorded a relatively uniform internal-body absorbed dose rate of 0.379 ± 0.010 mGy/day in H_2_O during the GCR phase of Artemis I. With a GCR radiation quality factor of 3.1 (as measured by the HERA HSU2 detector in the Orion crew cabin during the same time period), the dose rate is found to be 1.18 ± 0.03 mSv/day, which can be scaled to effective dose rates of ∼2.01 mSv/day during solar minimum and ∼0.615 mSv/day during solar maximum using the Badhwar-O’Neill 2020 GCR flux model (*52*, *53*). Based on these values for GCR exposure during nominal operations and the SPE dose results for unvested versus vested astronauts provided in Table 1, it is predicted that wearing the AstroRad vest inside the Orion crew cabin would increase an astronaut’s permissible time in space by between 59 and 193 days if exposed to a single August 1972–like SPE and by between 40 and 131 days if exposed to a single October 1989–like SPE, depending on the phase of the solar cycle (*36*, *53*). Such significant career extensions may be indispensable due to the immense financial investment associated with training astronauts and could be critical in enabling long-duration Mars missions.

The current NASA Space Flight Human-System Standard also provides guidelines for mission planning, which include ensuring that astronauts shall not exceed an effective dose of 250 mSv due to exposure to an SPE with an identical spectrum to that of the October 1989 event (*51*). It was found, for the simulations with the whole-body phantom, that an unvested astronaut inside the Orion crew cabin would be expected to come close to this threshold during a large SPE (204.2 mSv for an August 1972–like SPE and 219.0 mSv for an October 1989–like SPE). This study demonstrates that the AstroRad vest is expected to function well in reducing the risk of exceeding NASA’s current effective dose limits in the case of a large SPE, reducing effective dose to the whole-body phantom to 85.3 mSv for an SPE with the spectral shape of the August 1972 event and 138.2 mSv for an SPE with the spectral shape of the October 1989 event.

It is conceivable that a future SPE may even be substantially stronger than the August 1972 or October 1989 events, as the spectra of SPEs have only been reliably measured in the last century (*54*). Concentrations of cosmogenic nuclides found in tree rings and ice cores give evidence of years with integrated fluence exceeding measurements from the space era by one to two orders of magnitude (*55*–*59*). If these fluences were associated with single events or continuous event series, such exposures would expose crew members to doses on the order of several grays and introduce the risk of acute radiation sickness, even within the protection of a strongly shielded vehicle such as Orion. It is known from studies of clinical exposures and radiation incidents/accidents that bone marrow failure is the primary cause of death for acute radiation exposures up to as high as 6 Gy, beyond which damage to gastrointestinal tissue begins to manifest as an additional contributing factor to mortality (*60*). Due to the regenerative nature of bone marrow, as little as 2.5% of the entire body’s bone marrow must be spared in order for an individual to experience a significantly reduced likelihood of developing hematopoietic ARS (*61*, *62*). This observation is used in standard clinical practice, when cancer patients who undergo total body irradiation at typically lethal dose levels are able to survive after being given transplants containing 5% of the donor’s nucleated marrow cells (*63*). Based on the analysis in this study, together with the established human bone marrow lethality curve (*64*), a hypothetical SPE with the same spectral shape as the October 1989 SPE, but scaled up in intensity to deliver an average equivalent dose of 6 Gy-Eq to the RBM of an unshielded astronaut inside the Orion crew cabin [using NASA’s recommended RBE of 1.5 for protons >2 MeV; (*51*)], would be predicted to eliminate all but 2.1% of RBM, below the accepted threshold for survival from hematopoietic ARS. In contrast, wearing the AstroRad vest under the same conditions would be predicted to increase the surviving RBM fraction to 5.5%, surpassing the 2.5% survival threshold and even the 5% clinical target (fig. S7).

Last, to evaluate the benefits of a targeted approach to space radiation protection, a set of supplemental simulations were run using a full-body HDPE suit of uniform thickness and equivalent mass to the MARE AstroRad vest. Broadly, this shielding configuration resulted in substantially decreased protection compared to the vest, with the exception of the organs and tissues in the head, neck, and extremities, which are not as heavily weighted in contribution to effective dose. Furthermore, such a full-body suit would be much more difficult to don than a vest and would also be expected to impede movement and reduce ergonomics.

Although, thus far, the vest has been compared to an unshielded astronaut inside the Orion crew cabin in standard configuration, a radiation storm shelter may be constructed by Artemis crew within the spacecraft during an SPE. The original design for the storm shelter, developed by Lockheed Martin, uses the heavily shielded cargo bay area of the vehicle and readily available cargo supplies to reduce the dose equivalent during an October 1989–like SPE by 36%, per internal NASA analysis using 3DHZETRN, which was similarly validated using Artemis I data and a full CAD model of Orion (*36*, *65*). This reduction is very close to that which was determined to be provided by the vest in this work. However, the Cargo Bay shelter design significantly restricts crew mobility, and an updated, Whole Cabin shelter design was developed by NASA, which involves the crew mounting readily available cargo supplies to the least shielded wall of the Orion crew cabin. This allows for crew use of the cabin at the cost of reducing the effectiveness of the storm shelter by almost half. A central tenet in the field of radiation protection is the principle that radiation exposure should be kept to levels as low as reasonably achievable (ALARA). Viewed from this perspective, it can be seen that there are costs and benefits to each shielding approach: (i) The AstroRad vest (as flown on Artemis I) permits crew mobility and provides maximal protection at the cost of 26 kg of dedicated shielding mass, (ii) the Cargo Bay storm shelter provides maximal protection without additional mass at the cost of comfort and mobility, and (iii) the Whole Cabin storm shelter permits crew mobility without additional mass at the cost of protection. However, all three shielding approaches control exposure to the October 1989 SPE to below the 250-mSv NASA SPE effective dose limit (*51*).

In light of these considerations, a wearable protective vest such as AstroRad presents an attractive ALARA solution for non–mass-limited missions or for missions using large functional habitation areas that are less shielded and cannot be as easily or effectively reconfigured as Orion. Examples include many of the next-generation spacecraft, including large-volume vehicles such as the SpaceX Starship, inflatable habitation modules (*66*), and large space station modules in the style of Skylab that are currently being designed by Vast Space and Gravitics, Inc. (*67*, *68*). Alternative solutions to address mass limitations, which are currently being explored, would be for AstroRad vest panels to be assembled in space using water bags, a concept that was described previously in a StemRad patent (2019, filed in 2016) (*69*) and shown to be functionally feasible (but not tested in terms of radiation shielding efficacy) by the Italian Space Agency onboard ISS in November 2017 (*70*–*72*), or via additive manufacturing of recycled plastic waste (*73*).

In this work, we have shown based on validated extrapolation of empirical data from Artemis I that in the event of a major SPE, wearable radiation shielding such as the AstroRad vest can significantly reduce radiation exposure, even within an inherently heavily shielded vehicle such as the Orion spacecraft, helping to enable a safe and sustainable human presence beyond LEO.

## MATERIALS AND METHODS

### Design and construction of the MARE AstroRad vest

The AstroRad vest used in the MARE experiment was custom-made for the Zohar phantom. To generate the thickness distribution of the vest shielding, foci of protection were designated at the geometric centers of AstroRad target organs (stomach, colon, lungs, breast glandular tissue, iliac red bone marrow, and ovaries) using the XCAT digital model of the phantom. For each point along the external surface of the phantom, ray tracing was used to determine the amount of tissue self-shielding (in units of grams per square centimeter) along a straight path to each focus of protection (*12*). This produced one value of areal density of tissue self-shielding per focus of protection at each point on the surface of the phantom, of which the lowest value was selected, representing the areal density between the surface and the nearest focus of protection, and then rounded to the nearest 1 g/cm^2^. Beyond an areal density of 22 g/cm^2^ of hydrogenous shielding, the effects of additional shielding diminish significantly (*74*). Thus, in order to produce the complementary shielding thickness distribution of the AstroRad vest (Fig. 2A), the rounded tissue self-shielding values on the surface of the phantom were subtracted from 22 g/cm^2^, and the resulting quantities were uniformly scaled by a factor of 325 mm^3^/g. This final scaling was necessary to produce a vest with practical mass and thickness properties. The maximum thickness of the vest was limited to 65 mm, while the minimum thickness was set at 13 mm for the sake of structural continuity.

### Active dosimeters

The radiation detectors used in the DLR M-42 instruments are Hamamatsu S3590-19 planar silicon PIN photodiodes with a sensitive area of 11.0 mm by 11.1 mm and a thickness of 300 μm. For MARE, the energy deposition range of the M-42s was adjusted to between 35 keV and 20 MeV and the time resolution was set to record data every 5 min. The M-42 instruments include various additional built-in environmental sensors for quantities such as temperature and acceleration. The built-in accelerometers enabled the detectors to stay in “sleep-mode” until the moment of launch, thereby conserving battery power (*42*). The NASA CADs implement low-power-usage direct ion storage dosimeters (Mirion Technologies), enabling a long battery life on the order of several months, and report dose to H_2_O (*43*).

### Monte Carlo simulations

Monte Carlo radiation transport simulations were developed using GEANT4 version 10.7.3 with the “QBBC” physics list and run on an Oracle Cloud Infrastructure supercomputer using an AMD EPYC 7J13 processor with 128 CPU cores (*44*–*47*). Table S1 lists all simulations run for this study.

For the validation/calibration simulations, which were designed to replicate as closely as possible the experimental conditions of Helga and Zohar’s journey through the inner Van Allen belt aboard Artemis I, an isotropic radiation field representing the time-integrated omnidirectional proton and electron spectra for the Artemis I inner Van Allen belt crossing was calculated using the AE9/AP9 trapped radiation computational models (*50*). Helga and Zohar were individually simulated using the corresponding XCAT digital phantom, voxelized at a resolution of 2 mm. The chemical compositions for the CIRS proprietary tissue-equivalent resins corresponding to average soft tissue, average bone, cartilage, spinal cord, spinal disks, lung, brain, sinus, and breast were drawn from table A1 in International Commission on Radiation Units and Measurements (ICRU) Report 46 (*75*), while the densities were defined using values published by CIRS. The directional distributions of areal mass density and material composition for the hull of the Orion spacecraft, as viewed from seats 3 and 4, were determined based on a raytracing analysis of a manufacturing-quality Orion CAD model (*49*). To incorporate the internal M-42 detector systems, dosimeter holder frames, and the polyether ether ketone (PEEK) support rod, parallel GEANT4 geometries were used (*48*, *76*). Zohar was equipped with a CAD model of the AstroRad vest, generated using CT imaging data of the vest in the experimental configuration, and a steel antivibration belt. Each simulated M-42 detector featured a 3 cm–by–3 cm–by–300 μm silicon active area, artificially enlarged for improved statistics. The CAD detectors were modeled as pure water.

The SPE extrapolation simulations eliminated the “fixed orientation” constraint on the phantoms, accounting for random rotation of astronauts within the spacecraft, by modeling the spacecraft hull as a uniform shell, which varied in material density and composition according to the directional distribution of these variables in the spacecraft (Eq. 1 and Fig. 8, B and C). In addition, the input radiation field was swapped from the Artemis I inner Van Allen belt crossing spectrum to either the August 1972 SPE or the sum of the October 1989 SPEs. The 1972 SPE was modeled using the King parameterization (*77*), while the 1989 SPE was modeled using the Tylka parameterization (also known as the NASA design reference SPE) (*78*, *79*). For the ATOM phantom simulations, all of the MARE auxiliary hardware was also removed (Fig. 8A), and the phantoms were modeled using human tissue (per table A1 in ICRU Report 46) rather than tissue-equivalent resin (*75*). For the SPE simulations with the whole-body phantom, the ATOM phantom was replaced by a full-body median female XCAT phantom (height: 163 cm, weight: 62.0 kg), voxelized at a resolution of 4 mm (fig. S3) (*40*, *41*). The upper limbs of the phantom were positioned in the reference zero gravity neutral posture, as defined in figure 4.3-2 of the NASA Human Integration Design Handbook (*80*). Chemical compositions and densities of tissues and organs were again defined using table A1 in ICRU Report 46 (*75*).

### Biological dose calculation

Different species of radiation vary in their ability to cause biological damage, even for the same amount of physical dose deposited in tissue, due to differences in ionization density and distribution of energy deposition. This observation has led to various formulations of biological “dose equivalent,” in which the absorbed dose (in units of gray) is scaled relative to γ-ray exposures by a radiation quality factor dependent on the incident particle’s species and energy characteristics. In this work, the NASA formulation of the quality factor is used, as described by the NASA Space Cancer Risk (NSCR) model (version NSCR-2022), for which dose equivalent is given in units of sievert (*34*). Different organs and tissues also vary in their biological susceptibility to radiation. Thus, whole-body biological dose is typically expressed as a weighted sum of average dose equivalent in each tissue type, known as “effective dose” (also in units of sievert), for which we have also used the NASA-recommended tissue weights in this work (*33*).
