# Supplementary material for: First evaluation of wearable radiation protection for human deep space exploration, as flown on Artemis I
Source: Sci Adv. 2026 Aug 12;12(33):eadz1892. doi: 10.1126/sciadv.adz1892 (PMC13464478; doi:10.1126/sciadv.adz1892)
Supplement: Supplementary file 1 [file sciadv.adz1892_sm.pdf]

Science Advances
AAAS

Supplementary Materials for

First evaluation of wearable radiation protection for human deep space exploration, as flown on Artemis I

Jordan M. Houri *et al.*

Corresponding author: Jordan M. Houri, jordan@stemrad.com; Oren Milstein, oren@stemrad.com

Sci. Adv. **12**, eadz1892 (2026)
DOI: 10.1126/sciadv.adz1892

This PDF file includes:

Figs. S1 to S7
Tables S1 to S3

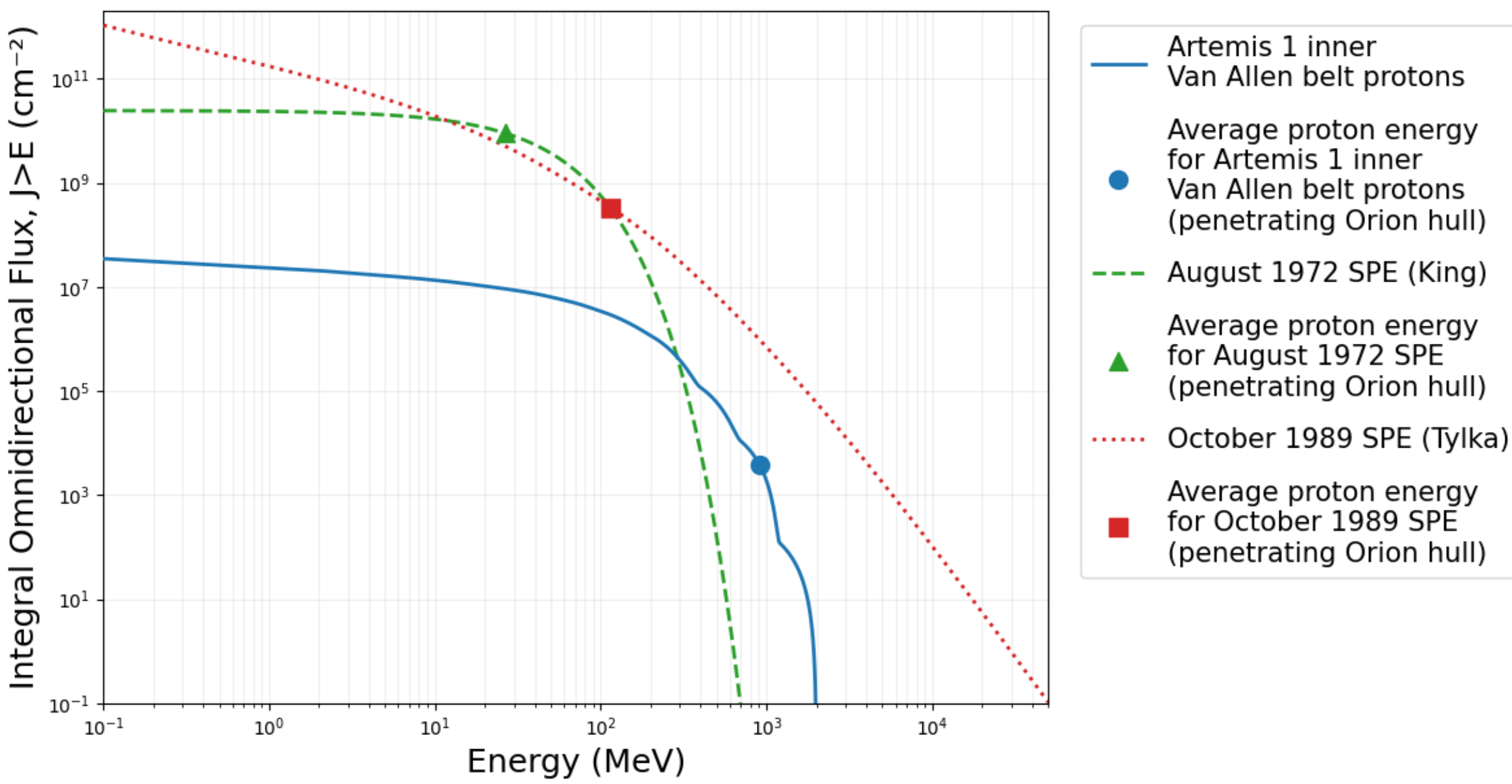

Fig. S1. Integral proton spectra for exposure scenarios analyzed/modeled in this study.
Integral proton spectrum for the Artemis I inner Van Allen belt transit modeled using AP9 (blue) compared to the King parameterization of the August 1972 solar particle event (SPE) integral proton spectrum (green dashed) and the Tylka parameterization of the October 1989 SPE integral proton spectrum (red dotted). The average residual energy of primary SPE protons following attenuation by the Orion spacecraft's hull is denoted for each spectrum, showing that, while the inner Van Allen belt spectrum has similar shape to that of an SPE, the average energy of inner Van Allen belt protons is substantially higher.

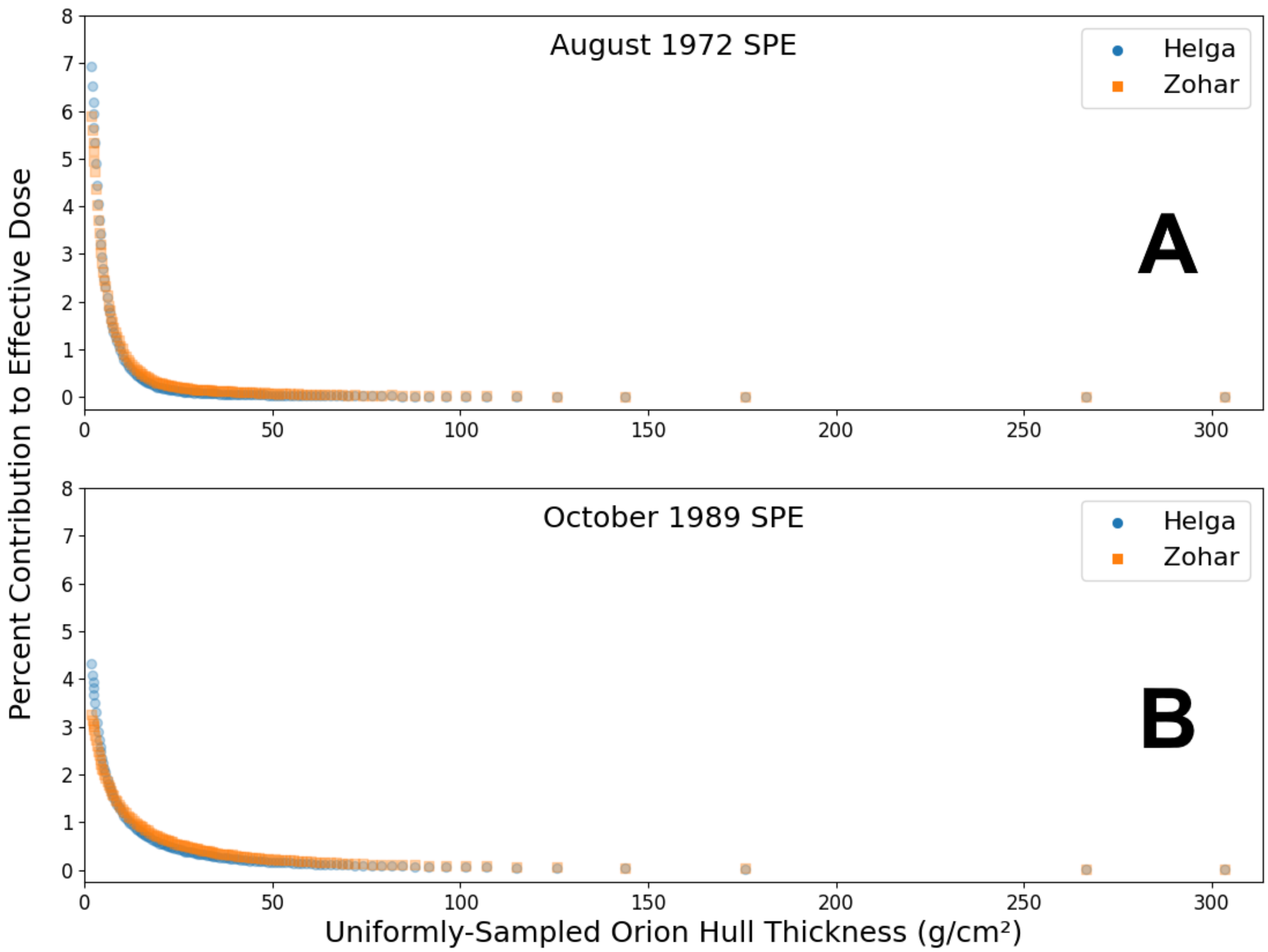

Fig. S2. Percent contribution to effective dose as a function of hull thickness.
Scatter plots showing the percent contribution to total effective dose for Helga and Zohar versus Orion hull thickness as exposed to the August 1972 solar particle event (SPE) spectrum **(A)** and the October 1989 SPE spectrum **(B)**, as calculated by the simulations in this study. Each data point is the result of one of 128 supercomputer simulations with uniformly-sampled hull thickness from the Orion spacecraft, cf. Figure 8 in the main text. It can be seen that the least-shielded regions of the spacecraft contribute overwhelmingly to radiation exposure.

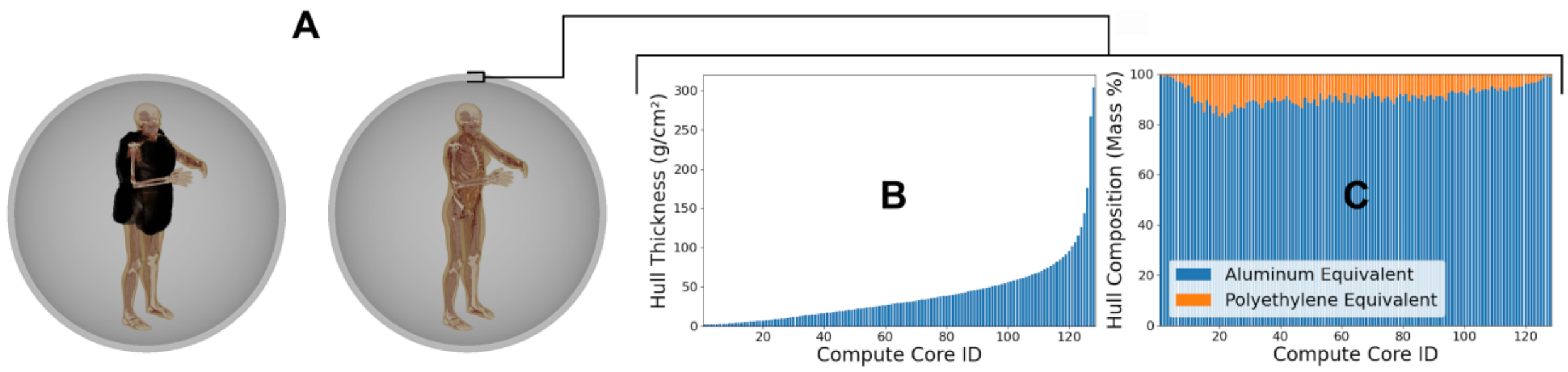

Fig. S3. Solar particle event (SPE) extrapolation phase simulation setup using the whole-body female XCAT phantom.
(A) SPE extrapolation phase simulation setup visualized in GEANT4 with spherical hull of uniform thickness for the whole-body female XCAT phantom wearing the AstroRad vest (left) and unvested (right). **(B)** Distribution of hull thicknesses (g/cm^2) versus compute core ID. **(C)** Hull material composition versus compute core ID.

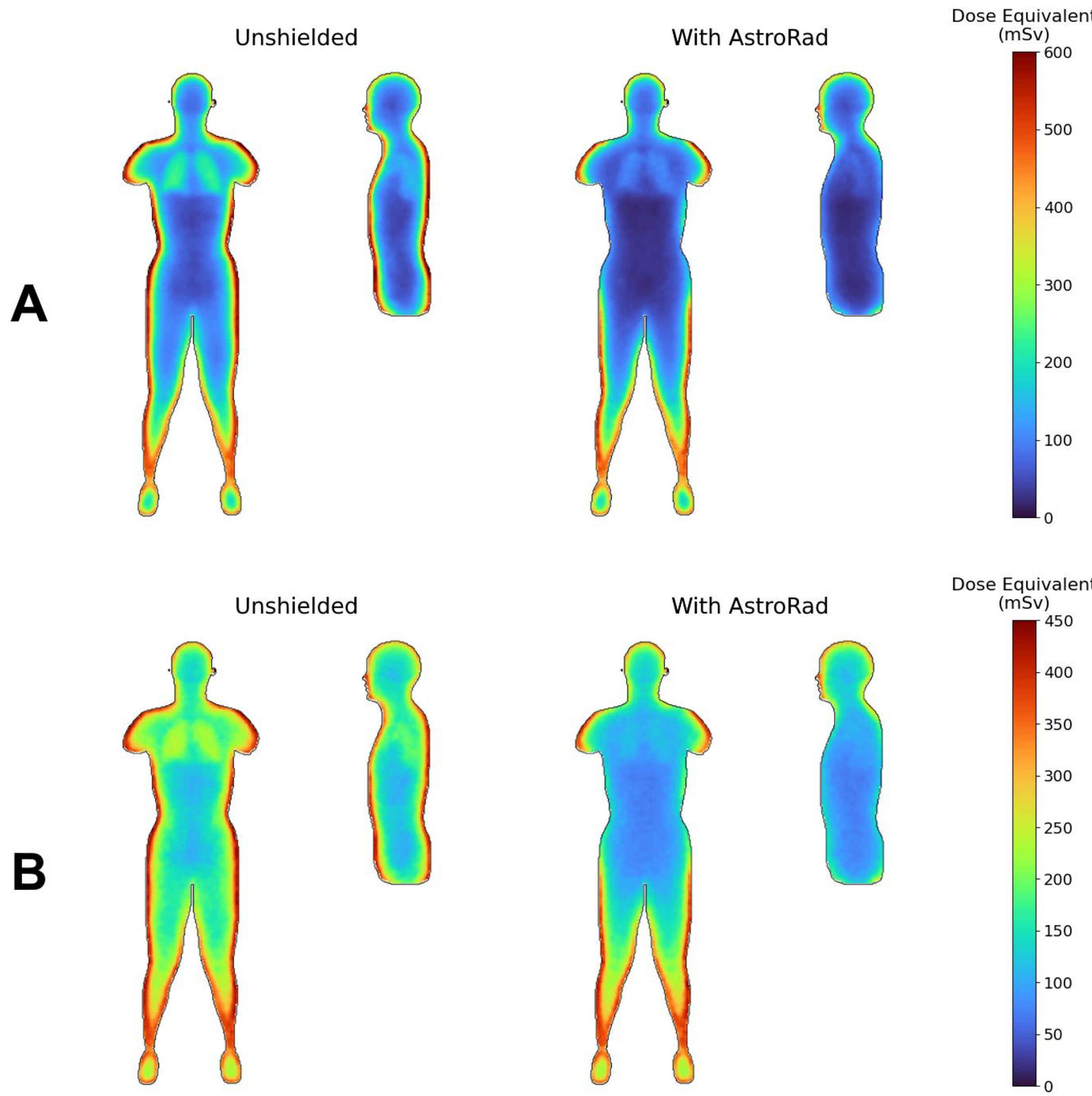

Fig. S4. Dose maps with and without AstroRad in solar particle event (SPE) conditions for the whole-body phantom.

Coronal and sagittal medial slices through the 3-dimensional NASA dose equivalent maps for the whole-body XCAT phantom both unvested (left) and wearing the AstroRad vest (right) as exposed to the August 1972 SPE **(A)** and the October 1989 SPE **(B)**. The arms are not visible in the coronal slices due to their position in front of the body. Note the different color scale between the 1972 SPE and 1989 SPE dose maps.

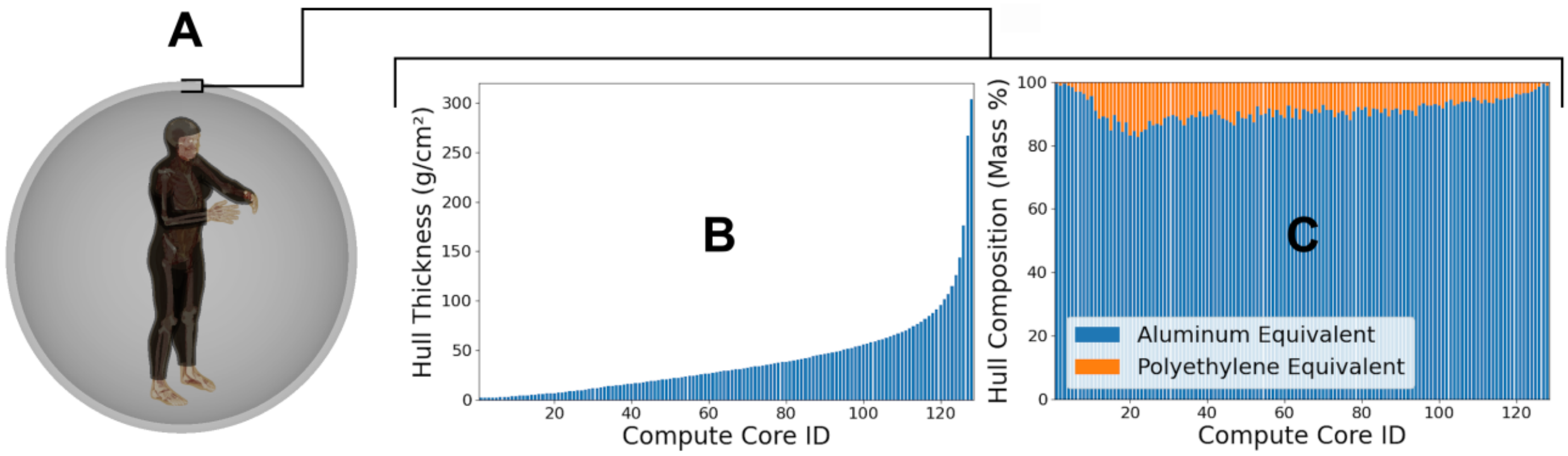

Fig. S5. Solar particle event (SPE) extrapolation simulation using a full body uniform high-density polyethylene (HDPE) suit instead of the AstroRad vest.
(A) Simulation setup visualized in GEANT4 with spherical hull of uniform thickness surrounding the whole-body female XCAT phantom wearing a full body uniform suit. **(B)** Distribution of hull thicknesses (g/cm^2) versus compute core ID. **(C)** Hull material composition versus compute core ID.

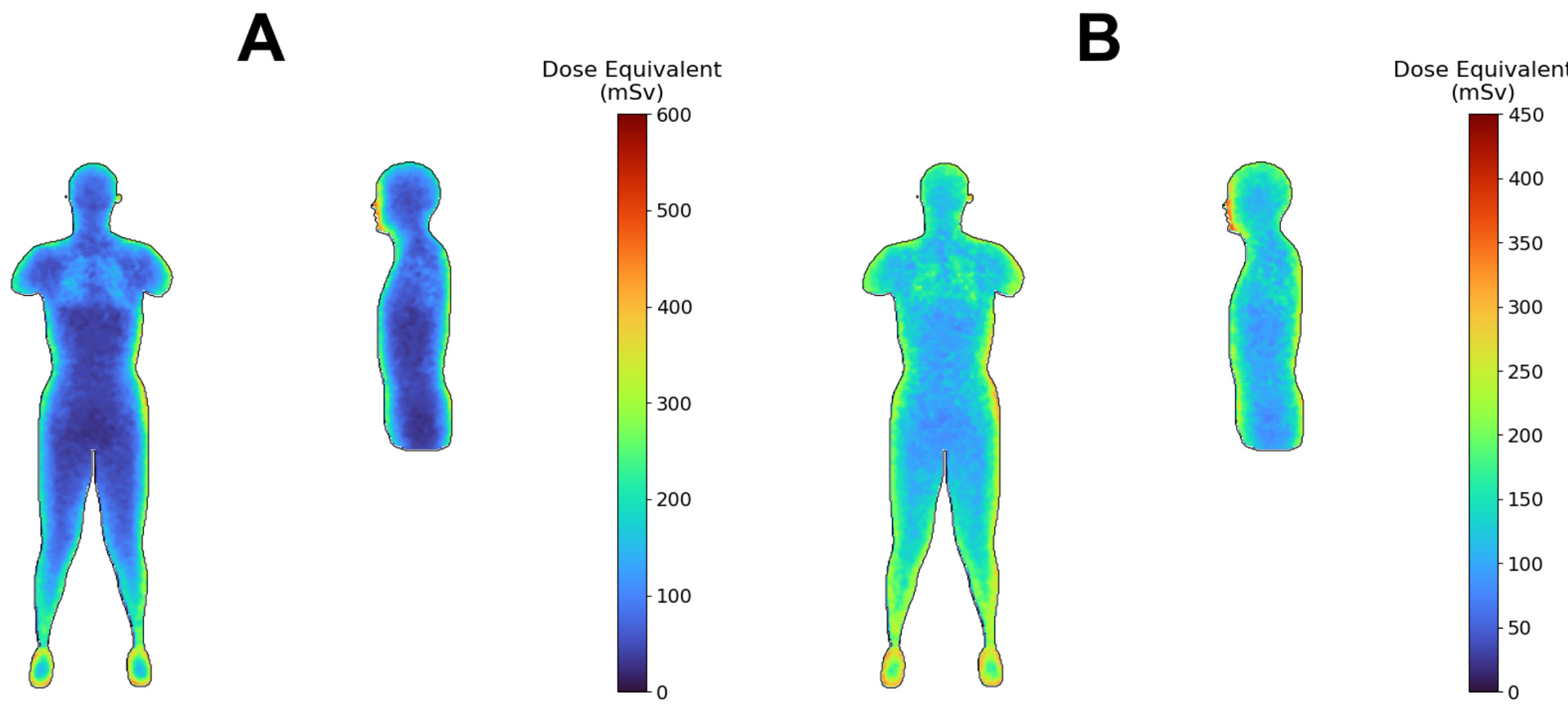

Fig. S6. Dose maps for protection by a full body uniform high-density polyethylene (HDPE) suit. Coronal and sagittal medial slices through the 3-dimensional NASA dose equivalent maps for the whole-body XCAT phantom wearing a full body HDPE suit of uniform thickness and the same mass as the MARE AstroRad vest as exposed to the August 1972 solar particle event (SPE) **(A)** and the October 1989 SPE **(B)**.

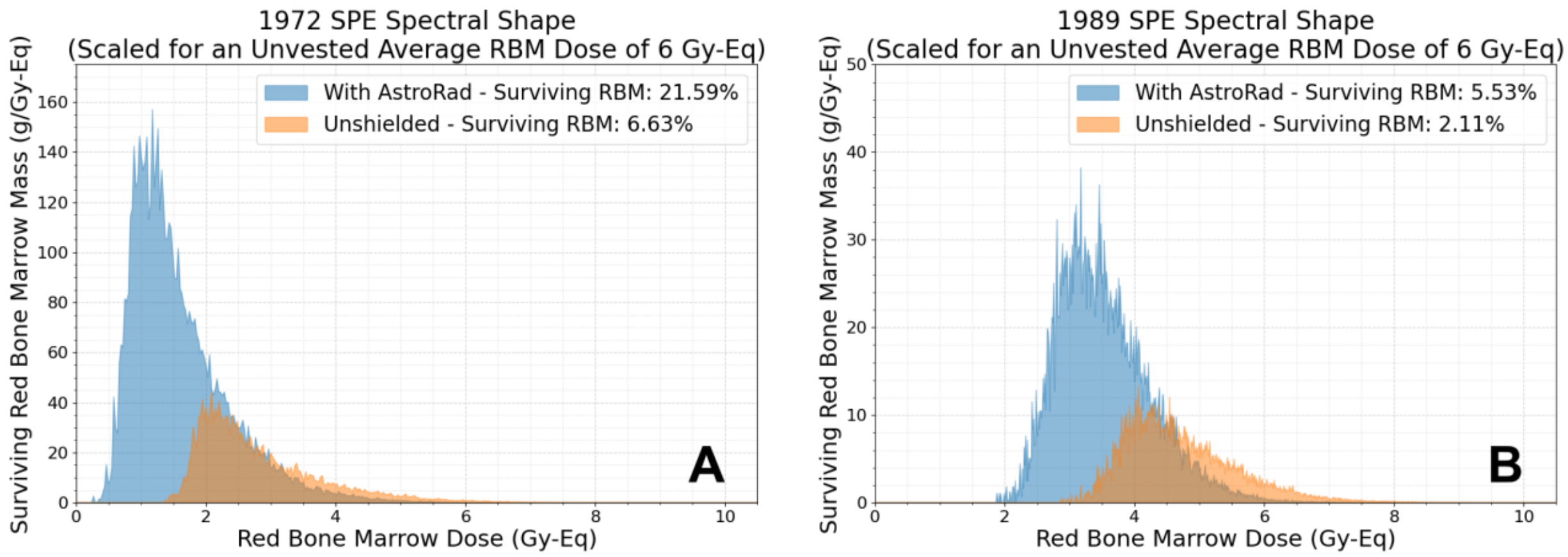

Fig. S7. Red bone marrow (RBM) survival histograms after scaling unvested RBM dose to 6 Gy-Eq. Plots showing the mass of RBM surviving as a function of received equivalent dose from the 1972 (**A**) and 1989 (**B**) solar particle event (SPE) spectra, scaled in magnitude such that the mean equivalent dose to the RBM of the unshielded whole-body phantom inside the Orion crew cabin would be 6 Gy-Eq (using a constant RBE of 1.5 for scaling of absorbed dose to equivalent dose). The orange curves represent surviving marrow in the unshielded case, while the blue curves represent surviving marrow with the AstroRad vest. Note the different Y-axis scale in each plot.

Table S1. Parameters for the validation/calibration simulations and prediction simulations

	Validation/Calibration		Prediction				
	Helga	Zohar	Unvested		With AstroRad		With Full-body HDPE Suit
Phantom Model	CIRS ATOM 702 Torso		CIRS ATOM 702 Torso (Helga)	Median Adult Female Whole-Body XCAT	CIRS ATOM 702 Torso (Zohar)	Median Adult Female Whole-Body XCAT	Median Adult Female Whole-Body XCAT
Hull Model	Shell with directional distribution of Orion areal density and composition		Uniform shell with variable areal density and composition				
Radiation Spectrum	Artemis I Inner Van Allen Belt		August 1972 SPE			October 1989 SPE	

Table S2. NASA dose equivalent for specific organs and tissues as calculated by the validation and SPE extrapolation simulations with color grading for dose reduction columns.
Reproduction of Table 1 in the main text with a color grading applied to the percent dose reduction columns (darker green representing greater reductions). For the solar particle event (SPE) extrapolation simulation percent dose reductions, the color scale corresponds to the average of the percent reduction values for the torso phantom and the whole-body phantom. Italicized values represent organs specifically targeted for protection by the AstroRad vest. For the SPE extrapolation simulations, results are provided for both the ATOM torso phantom and the whole-body phantom (in parentheses).

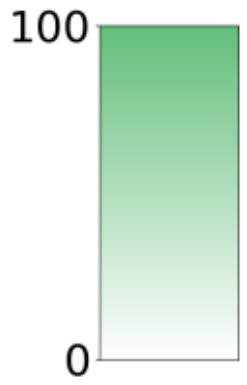

	NASA Dose Equivalent (mSv)								
	Artemis I Inner Van Allen Belt Transit			August 1972 SPE			October 1989 SPE		
	Helga	Zohar	Percent Reduction	Helga or (Whole-body unvested)	Zohar or (Whole-body w/ AstroRad)	Percent Reduction	Helga or (Whole-body unvested)	Zohar or (Whole-body w/ AstroRad)	Percent Reduction
Pelvic and Lumbar Red Bone Marrow	*1.04*	*0.80*	*22.7*	*77.6<br>(72.4)*	*33.0<br>(30.9)*	*57.5<br>(57.3)*	*136.8<br>(130.8)*	*94.8<br>(90.9)*	*30.7<br>(30.5)*
Breasts	*2.52*	*1.55*	*38.4*	*510.2<br>(520.3)*	*127.8<br>(129.7)*	*75.0<br>(75.1)*	*398.2<br>(404.6)*	*179.0<br>(181.3)*	*55.0<br>(55.2)*
Colon	*1.60*	*1.09*	*32.0*	*153.4<br>(136.2)*	*53.3<br>(49.3)*	*65.3<br>(63.8)*	*187.8<br>(176.1)*	*114.6<br>(109.0)*	*39.0<br>(38.1)*
Lungs	*1.76*	*1.34*	*24.0*	*288.8<br>(245.0)*	*111.5<br>(95.5)*	*61.4<br>(61.0)*	*284.9<br>(254.5)*	*170.7<br>(153.5)*	*40.1<br>(39.7)*
Gonads (Ovaries and Uterus)	*1.27*	*0.97*	*23.5*	*53.7<br>(52.7)*	*26.6<br>(26.2)*	*50.5<br>(50.2)*	*119.0<br>(115.9)*	*88.6<br>(86.6)*	*25.6<br>(25.3)*
Stomach	*1.53*	*1.17*	*23.5*	*142.8<br>(122.2)*	*47.8<br>(43.2)*	*66.5<br>(64.7)*	*185.9<br>(167.4)*	*112.6<br>(106.1)*	*39.4<br>(36.6)*
Spine	0.96	0.81	15.6	115.7<br>(93.7)	62.6<br>(54.0)	45.9<br>(42.4)	167.1<br>(144.1)	120.6<br>(105.8)	27.8<br>(26.6)
Bladder	1.39	1.02	26.6	85.7<br>(82.7)	36.1<br>(33.5)	57.9<br>(59.5)	140.8<br>(136.0)	97.9<br>(95.6)	30.5<br>(29.7)
Liver	1.49	1.00	32.9	140.3<br>(120.9)	50.0<br>(43.2)	64.4<br>(64.3)	184.7<br>(168.7)	114.3<br>(105.2)	38.1<br>(37.6)
Esophagus	1.23	1.04	16.0	97.6<br>(84.5)	54.5<br>(48.3)	44.1<br>(42.9)	166.0<br>(149.8)	122.9<br>(111.9)	26.0<br>(25.3)
Thyroid	1.59	1.40	12.0	195.2<br>(158.6)	135.7<br>(114.3)	30.5<br>(28.0)	222.3<br>(189.5)	179.6<br>(163.5)	19.2<br>(13.7)
Skin	2.11	1.52	28.0	838.1<br>(708.4)	365.5<br>(508.0)	56.4<br>(28.3)	552.1<br>(494.4)	298.4<br>(382.4)	46.0<br>(22.7)
Bone Surface	1.14	0.97	15.1	174.2<br>(172.9)	109.2<br>(151.8)	37.3<br>(12.2)	189.0<br>(195.8)	142.6<br>(176.7)	24.6<br>(9.8)
Salivary Glands	1.72	1.59	7.8	294.5<br>(223.2)	239.2<br>(194.7)	18.8<br>(12.8)	272.2<br>(224.1)	236.7<br>(202.2)	13.0<br>(9.8)
Heart	1.38	1.07	22.2	103.1<br>(93.2)	48.1<br>(40.7)	53.4<br>(56.3)	166.6<br>(153.2)	116.1<br>(106.2)	30.3<br>(30.7)
Brain	1.17	1.14	2.4	171.3<br>(143.0)	165.9<br>(140.1)	3.1<br>(2.0)	204.3<br>(184.3)	198.0<br>(180.6)	3.1<br>(2.0)
Eyes	1.80	1.73	3.8	283.8<br>(235.7)	273.1<br>(229.3)	3.8<br>(2.7)	267.4<br>(234.0)	259.2<br>(228.2)	3.0<br>(2.5)
Intestines	1.48	1.07	27.9	124.8<br>(112.8)	44.4<br>(42.0)	64.4<br>(62.7)	170.3<br>(160.3)	106.6<br>(102.2)	37.4<br>(36.2)

Kidney	1.14	0.85	25.7	105.3 (97.4)	42.7 (40.5)	59.5 (58.5)	161.3 (154.1)	105.8 (100.5)	34.4 (34.8)
Blood and RBM (Leukemia)	1.25	1.01	19.0	123.7 (105.9)	70.0 (64.6)	43.4 (39.0)	169.3 (154.6)	124.3 (116.8)	26.6 (24.5)
Oral Cavity	1.68	1.59	5.2	192.6 (101.8)	175.0 (97.2)	9.1 (4.5)	220.8 (156.8)	203.3 (150.2)	7.9 (4.2)
Pancreas	1.24	0.93	25.5	65.6 (59.5)	30.1 (27.3)	54.1 (54.2)	136.8 (130.8)	95.4 (91.5)	30.3 (30.0)
Spleen	1.31	1.05	19.6	152.1 (135.3)	51.6 (49.5)	66.1 (63.4)	193.3 (179.4)	116.0 (110.7)	40.0 (38.3)
**Total NASA Effective Dose**	**1.61**	**1.19**	**25.8**	**222.3 (204.2)**	**87.5 (85.3)**	**60.7 (58.2)**	**233.5 (219.0)**	**143.6 (138.2)**	**38.5 (36.9)**

Table S3. Protection provided by the AstroRad vest compared to a uniform full body suit. Percent improvement in protection is defined as the percent change in dose reduction provided by the AstroRad vest as compared to that provided by the full body suit. Italicized values represent organs specifically targeted for protection by the AstroRad vest. A color grading (ranging from -100% to 100%) is applied to the percent change in protection columns, with red representing decreases in protection and green representing increases in protection compared to the full body suit.

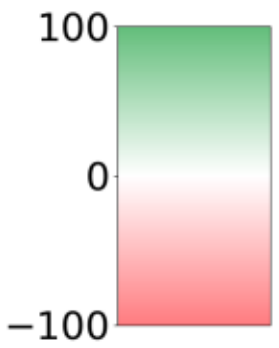

	NASA Dose Equivalent (mSv)							
	August 1972 SPE				October 1989 SPE			
	Unshielded	With AstroRad	Uniform Full Body Suit	Percent Improvement in Protection Using Selective Shielding	Unshielded	With AstroRad	Uniform Full Body Suit	Percent Improvement in Protection Using Selective Shielding
Pelvic and Lumbar Red Bone Marrow	*130.8*	*90.9*	*110.1*	*92.6*	*72.4*	*30.9*	*49.5*	*81.5*
Breasts	*404.6*	*181.3*	*265.5*	*60.5*	*520.3*	*129.7*	*271.7*	*57.1*
Colon	*176.1*	*109.0*	*134.7*	*62.1*	*136.2*	*49.3*	*81.2*	*57.9*
Lungs	*254.5*	*153.5*	*176.1*	*28.8*	*245.0*	*95.5*	*141.7*	*44.7*
Gonads (Ovaries and Uterus)	*115.9*	*86.6*	*99.9*	*83.3*	*52.7*	*26.2*	*31.6*	*25.3*
Stomach	*167.4*	*106.1*	*127.9*	*55.3*	*122.2*	*43.2*	*74.0*	*64.1*
Spine	144.1	105.8	117.4	43.1	93.7	54.0	59.0	14.3
Bladder	136.0	95.6	106.6	37.5	82.7	33.5	52.8	64.2
Liver	168.7	105.2	128.8	59.1	120.9	43.2	71.0	55.5
Esophagus	149.8	111.9	115.0	8.8	84.5	48.3	53.2	15.8
Thyroid	189.5	163.5	162.6	-3.5	158.6	114.3	114.8	1.2
Skin	494.4	382.4	266.8	-50.8	708.4	508.0	315.8	-49.0
Bone Surface	195.8	176.7	146.8	-60.9	172.9	151.8	114.4	-63.9
Salivary Glands	224.1	202.2	179.6	-50.8	223.2	194.7	155.6	-57.9
Heart	153.2	106.2	123.5	58.3	93.2	40.7	60.2	59.2
Brain	184.3	180.6	147.0	-90.1	143.0	140.1	95.9	-93.9
Eyes	234.0	228.2	215.1	-69.1	235.7	229.3	223.2	-49.3
Intestines	160.3	102.2	126.9	73.8	112.8	42.0	70.8	68.5
Kidney	154.1	100.5	119.9	56.6	97.4	40.5	65.4	77.9
Blood and RBM (Leukemia)	154.6	116.8	122.6	18.3	105.9	64.6	68.6	10.7
Oral Cavity	156.8	150.2	138.6	-63.5	101.8	97.2	86.8	-69.2
Pancreas	130.8	91.5	100.5	29.8	59.5	27.3	40.7	71.6
Spleen	179.4	110.7	140.1	75.0	135.3	49.5	90.0	89.6
**Total NASA Effective Dose**	**219.0**	**138.2**	**156.5**	**29.2**	**204.2**	**85.3**	**115.0**	**33.3**
